# Hybrid rule-based botnet detection approach using machine learning for analysing DNS traffic

**DOI:** 10.7717/peerj-cs.640

**Published:** 2021-08-13

**Authors:** Saif Al-mashhadi, Mohammed Anbar, Iznan Hasbullah, Taief Alaa Alamiedy

**Affiliations:** 1 National Advanced IPv6 Centre, Universiti Sains Malaysia, Penang, Malaysia; 2 Electrical Engineering, University of Baghdad, Baghdad, Baghdad, Iraq; 3 ECE Department- Faculty of Engineering, University of Kufa, Kufa, Najaf, Iraq

**Keywords:** Botnet detection, DNS analysis, Rule-based technique, Machine learning, Network security

## Abstract

Botnets can simultaneously control millions of Internet-connected devices to launch damaging cyber-attacks that pose significant threats to the Internet. In a botnet, bot-masters communicate with the command and control server using various communication protocols. One of the widely used communication protocols is the ‘Domain Name System’ (DNS) service, an essential Internet service. Bot-masters utilise Domain Generation Algorithms (DGA) and fast-flux techniques to avoid static blacklists and reverse engineering while remaining flexible. However, botnet’s DNS communication generates anomalous DNS traffic throughout the botnet life cycle, and such anomaly is considered an indicator of DNS-based botnets presence in the network. Despite several approaches proposed to detect botnets based on DNS traffic analysis; however, the problem still exists and is challenging due to several reasons, such as not considering significant features and rules that contribute to the detection of DNS-based botnet. Therefore, this paper examines the abnormality of DNS traffic during the botnet lifecycle to extract significant enriched features. These features are further analysed using two machine learning algorithms. The union of the output of two algorithms proposes a novel hybrid rule detection model approach. Two benchmark datasets are used to evaluate the performance of the proposed approach in terms of detection accuracy and false-positive rate. The experimental results show that the proposed approach has a 99.96% accuracy and a 1.6% false-positive rate, outperforming other state-of-the-art DNS-based botnet detection approaches.

## Introduction

Nowadays, especially during the global COVID-19 pandemic, there is no longer a debate that the Internet has become a core element of our daily life. Today’s Internet is about online presence, e-learning, social media, e-banking, work from home, online shopping, Internet of Things, and cloud computing (Stevanovic et al., 2012; Nozomi Networks Labs, 2020; Lallie et al., 2020). Unfortunately, Internet resources are continuously under threat by malicious actors, whether individual or organised entities. The botnet is now one of the most preferred tools by malicious actors for sophisticated cyber attacks. As a result, it is considered one of the critical threats to Internet users’ security and privacy (Nozomi Networks Labs, 2020).

There are two main motives for building and operating botnets: financial gain by offering botnets for hire for attacks and crypto mining and politics for hacktivism or nation-states. The services provided by the botnets vary from the crypto-mining campaign and intelligence gathering to anonymised large-scale cyber attacks (Almutairi et al., 2020).

**Figure 1:**
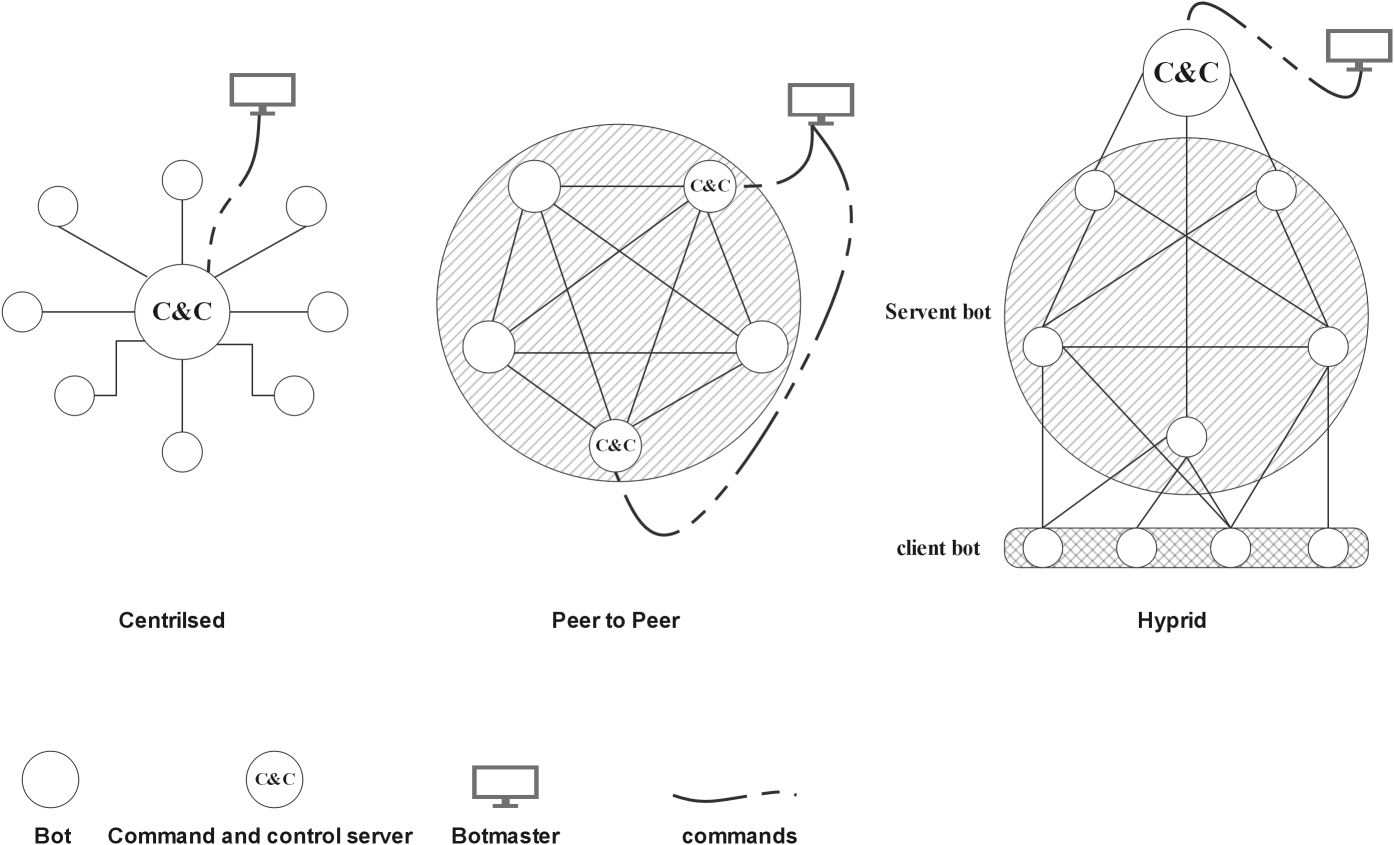
Botnet communication architecture.

A botnet comprises a network of malware-infected computing devices (Abu Rajab et al., 2006). A malware transforms compromised computing devices into robots (bots) controlled remotely by the attacker, known as a botmaster, without end-users knowledge (Asadi et al., 2020). Botmasters hide their location and avoid detection of law enforcement entities by controlling and initiating botnet attacks *via* the Internet through command and control (C&C) servers using various communication techniques (Almutairi et al., 2020). Figure 1 shows the botnet communication architecture.

**Figure 2:**
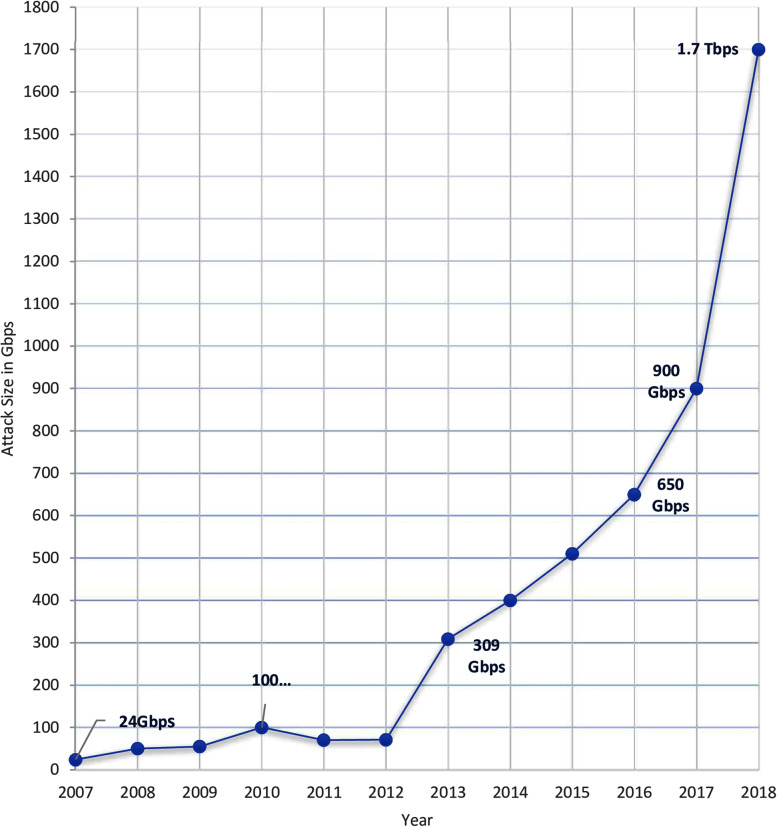
Size of Botnet DDoS attacks over 10 years (Morales, 2018).

Some of the botnet attacks include Distributed Denial of Service (DDoS), sending spam email, ransomware (Gu et al., 2013; Alomari et al., 2016), or phishing emails (Karim et al., 2014), and stealing sensitive data that could be used for further attacks. Even though there are different approaches to mitigate botnet attacks, since its first appearance in 1993 (Silva et al., 2013), the number of botnet attacks has been growing steadily. The 10-year trend of the size of botnet-based DDoS attacks (Morales, 2018) in Fig. 2 clearly shows that there is a marked increase from 2007 (24 Gbps) to 2018 (1.7 Tbps). Similarly, the Symantec Internet Security Threat Report (Symantec, 2018) reported a 62% increase in botnet activities in 2018 compared to the previous year.

Initiating and coordinating attacks require all members (bots) to be connected with each other and the C&C servers. This interconnectedness is fundamental for the botnet lifecycle (Khattak et al., 2014), which allows the botnet members to receive new commands and synchronise their actions.

There are three types of botnet communication architectures according to their communication topology: centralised (client-server), decentralised (peer-to-peer), and hybrid (Silva et al., 2013; Negash & Che, 2015). The bots in the centralised category connect to the C&C using IRC, HTTP, or DNS protocols to obtain instruction and update their status (Silva et al., 2013; Negash & Che, 2015). Table 1 provides a detailed comparison between the three botnet communication architectures.

**Table 1:** Comparison of botnet communication architectures.

Architecture	Description	Pro	Cons
Centralised	Bots are connected, get instruction and centrally update their status with the C&C using IRC, HTTP or DNS protocols (Silva et al., 2013)	Easy to construct and manage by attackers	A single failure point
Peer to Peer(P2P)	It is similar in technique to the P2P file-sharing system, where the bot has dual behaviour; it can act as a botmaster of C&C server to send commands and act like a typical bot when receiving the command from other bots (Al-Mashhadi et al., 2019). P2P is constructed so that each bot communicates with nearby bots in its system to organise a cluster. Example P2P botnets include GameOver Zeus, Sality	Immune to shut down (Singh, Singh & Kaur, 2019)	Managing difficulties due to the required routing protocols (Acarali et al., 2016)
Hybrid	It is a combination of P2P and centralised architecture, taking advantage of both (Khattak et al., 2014; Khan et al., 2019). In this architecture, the C&C server is central and consists of many P2P organised bots that forward the command to the server bots in a hierarchical manner. Besides, the botmaster uses proxy bots between their machine and the botnet, with each bot act as a servant transmitting commands to the bots that they compromised (Wang, Sparks & Zou, 2010)	More resistant to taking down this structure than the previous ones. It also provides profit for botmasters by allowing renting part of their botnet to provide different attack services	This architecture faces higher latencies in commands and control propagation than P2P, but they are very immune to downstream efforts since only a minor portion of the botnet will be affected if the C&C server has been shut down (Khattak et al., 2014)

The bots connect with the C&C server using pre-programmed static IP addresses of the C&C server within the malware codes or algorithm-generated domain names (Cantón, 2015).

It is possible to detect different types of botnet architecture by analysing DNS communication traffic, regardless of the communication architecture used (centralised, decentralised, or hybrid).

**Figure 3:**
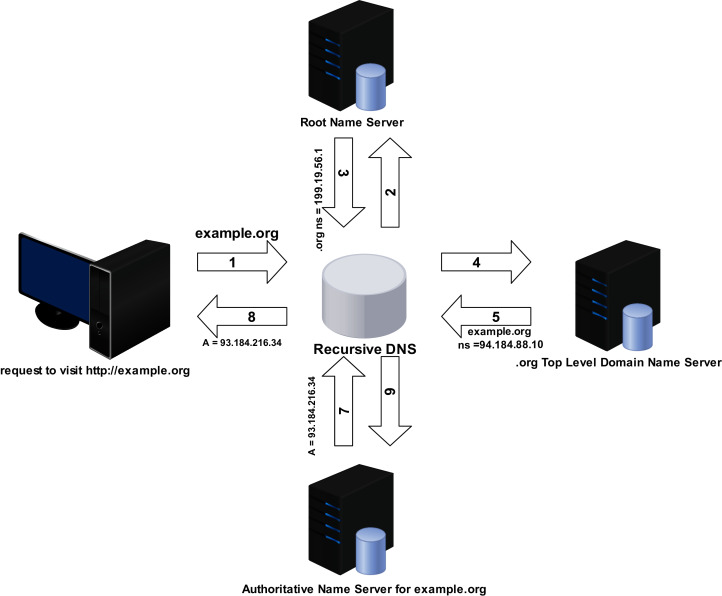
DNS resolving process.

DNS is all about resolving queries to map a domain name hierarchically to its corresponding IP address, similar to a phone book that catalogues human-readable domain names (URLs) and their related computer-readable IP address formats (numeric). Figure 3 illustrates the operation of domain name resolution.

DNS is an essential Internet service that cannot be disabled or blocked using firewalls without incapacitating the network functionality. For this reason, some botmasters rely on the DNS protocol for botnet communication (Mockapetris, 1987). Botmasters avoid detection by using dynamic DNS strategies that constantly and rapidly change domain names and their associative IP addresses. Two popular dynamic DNS techniques are fast-flux (Holz et al., 2008) and domain-flux (Yadav & Reddy, 2012).

**Figure 4:**
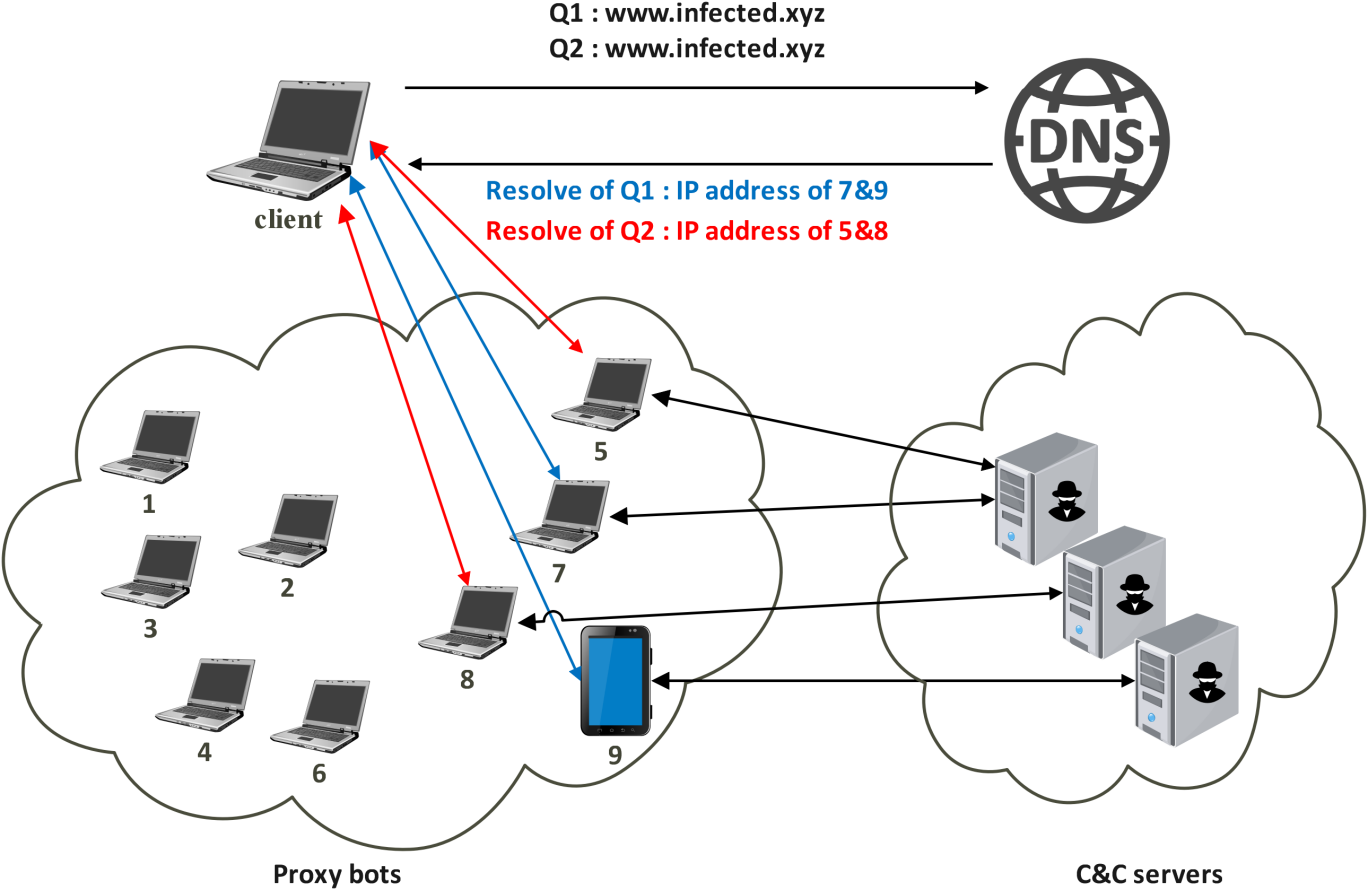
Typical fast-flux domain resolution.

As shown in Fig. 4, fast-flux is a technique that regularly assigns several IP addresses to the same domain name. The fast-flux approach is often used for legit purposes, such as load balancing by content delivery network operators (Yadav & Reddy, 2012). On the other hand, the domain-flux method is carried out by dynamically generating pseudo-random domains using the Domain Generation Algorithm (DGA).

**Figure 5:**
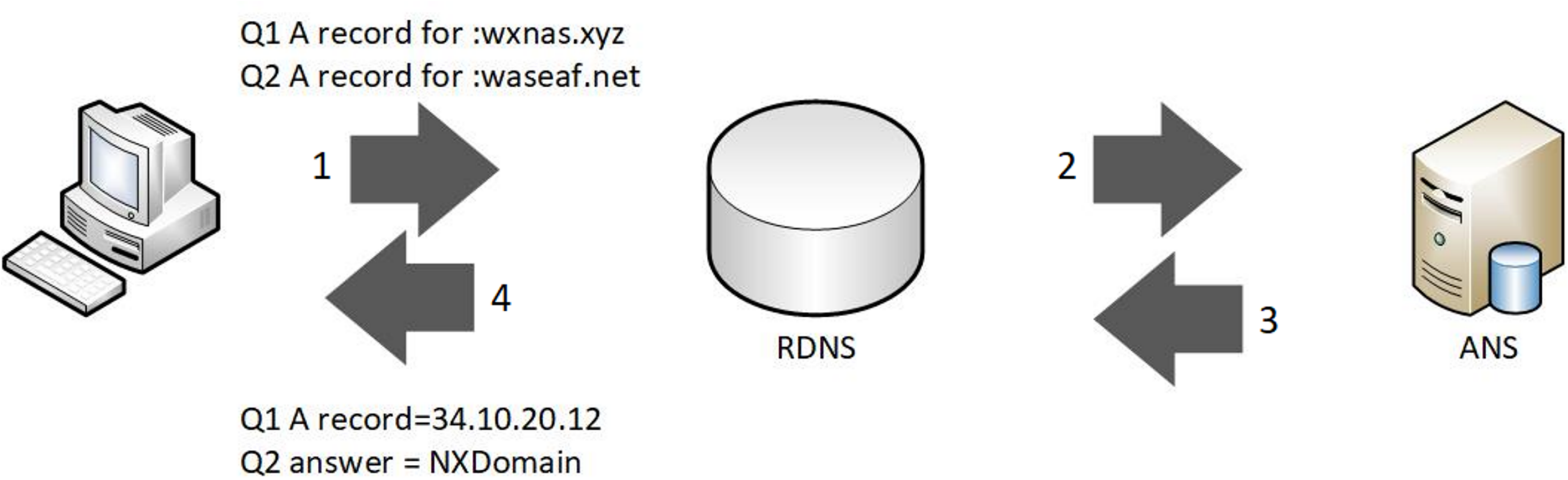
Typical procedure for DGA DNS resolution.

The DGA has several specific characteristics, as shown in Fig. 5. Firstly, there is no hardcoded domain name on the C&C server, making it unpredictable (Zago, Gil Pérez & Martínez Pérez, 2019). Secondly, the botmaster could use DGA as a fail-safe or backup channel when the primary communication channel fails (Stone-Gross et al., 2011). The Zeus worm (Luo et al., 2017) is one of the worms that employs DGA.

Combining fast-flux and DGA techniques allows constant modification of the C&C’s IP address and domain name to avoid detection (Zhou et al., 2013).

Although such techniques are complex, they are popular because they maintain the communication channel open and undetected by using dynamic but somewhat secret domain names. Examples of botnets that use the DGA technique to avoid detection are Necurs and Conficker. A Conficker bot generates up to fifty thousand new unique domain names daily but only using 500 of them for communication purposes. On the other hand, the Necurs bot systematically generates 2,048 new domains through an algorithm (Antonakakis & Perdisci, 2012).

**Figure 6:**
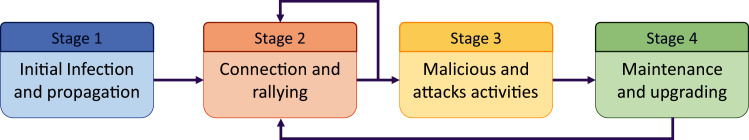
Botnet life—cycle.

The evasive techniques to control botnets generate abnormal traffic patterns throughout the botnet lifecycle phases. These patterns can be used to detect botnets. The botnet lifecycle could be broken down into four phases, as listed and illustrated in Fig. 6.

- **Initial infection and propagation phase:** In this phase, bot malware aims to infect Internet-facing devices, such as cell phones, personal computers, smart devices, and even CCTVs. The attacker has many tools and techniques at his disposal to identify exploitable vulnerabilities to gain access and control the targeted host. Some strategies include social engineering, spam, and phishing. Once a vulnerability is found and successfully exploited, the bot would connect to a remote server (botmaster) to download and install all required software to control the host device (Al-Mashhadi et al., 2019).
- **Connection and rallying phase:** In this phase, the bot tries to find and connect to the C&C server and other bots. The communication occurs either *via* the C&C server or a proxy server. The likelihood of exposure of the bot is the highest in this phase because this phase is repeated until a connection is established (Silva et al., 2013). Nevertheless, even with the risk of being exposed and discovered, the DNS lookup query is still widely used in the botnet connection phase since it is the most flexible botnet communication method (Manasrah et al., 2009).
- **The Malicious and attack phase:** The botmaster instructs the bots to perform nefarious activities, such as distributing malicious software or sending spam emails. Bots can also perform disruptive attacks, such as a DDoS attack (Da Luz, 2014).
- **The Maintenance and upgrading phase:** Bots remain idle while waiting for new commands from the botmaster. These commands might include new targets, update their behaviour, or instruction for new malicious activities. The botmaster will uphold the bots as long as possible by continuously upgrading them to avoid detection, enhancing propagation vectors with potential threats and methods or updates, and patching errors in scripts (Zeidanloo et al., 2010).

Some traits and data trails exist throughout the botnet life cycle or botnet communication despite employing evasive techniques. Examples of DNS data trails include domain names, resource code, DNS responses, DNS queries, and timestamps. Such DNS data trails’ availability provides security researchers with ways to detect botnets and their C&C servers (Stevanovic et al., 2012; Luo et al., 2017).

Given the discussion above, our research question is as follows: Can we increase botnet detection accuracy by combining two machine learning algorithms to analyse DNS data trails and the significant DNS-related features and rules that contribute to botnet detection?

This study’s goal is to enhance DNS-based botnet detection accuracy. The contributions of this paper are (i) new features derived from basic DNS features using Shannon entropy and (ii) a hybrid rule-based model for botnet detection using a union of JRip and PART machine learning classifiers. Finally, the evaluation of the proposed approach uses different datasets with various evaluation metrics; and the results are compared with other existing methods.

The rest of this paper is organised as follows. The related literature and studies section presents the current related work. The Section “Related Literature and Studies” details the proposed approach framework. This study’s implementation environment is in Section “Materials & Methods”, and the Section “Results” is devoted to elaborating the result and discussion. Finally, the conclusion and future research directions in the Section “Conclusion” concludes this paper.

## Related literature and studies

**Figure 7:**
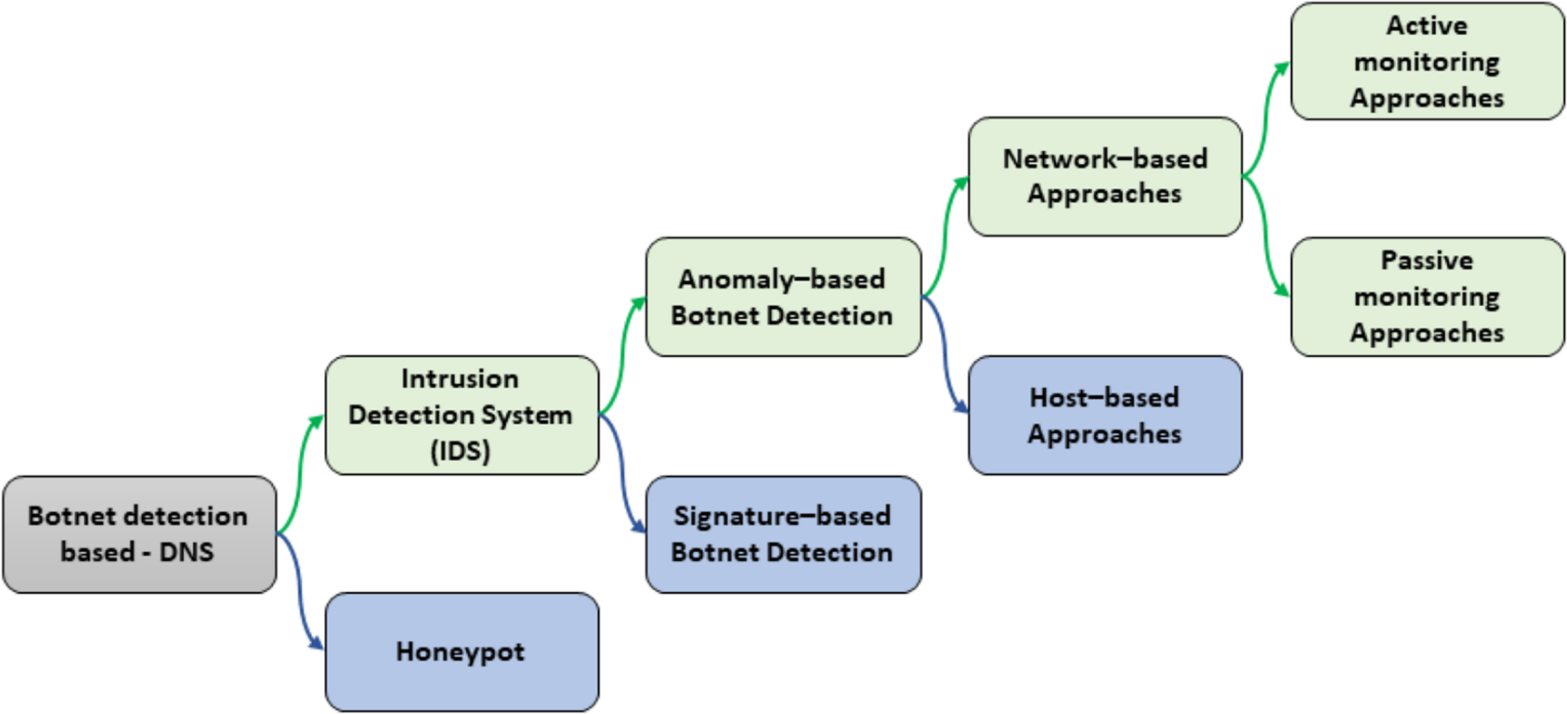
Taxonomy of Botnet detection based on DNS.

Currently, there are two main methods to detect DNS-based botnet: Honeypot and Intrusion Detection Systems (IDS) (Dornseif, Holz & Klein, 2004; Anbar et al., 2016). Figure 7 presents the taxonomy of the DNS-based botnet detection approaches.

### Honeypots

Honeypots are widely used for identifying and analysing the behaviour of botnet attacks. Honeypots are purposely designed to be vulnerable to botnet attacks to capture and gather as much data as possible on the botnet (Freiling, Holz & Wicherski, 2005). Honeypot also runs specialised software that attempts to match bots’ signatures and discovers the location of the botnet’s C&C server.

There are at least three types or levels of honeypots depending on the required level of bots information, the complexity of the study’s data, and the interaction level permitted to the attacker: low, medium, and high (Koniaris, Papadimitriou & Nicopolitidis, 2013; Nawrocki et al., 2016). A low-level honeypot or Low Interaction Honeypot (LIH) stores unauthorised communication with a limited attacker interaction; therefore, it is safer and easier to maintain than other honeypot types. A Medium Interaction Honeypot (MIH) provides more meaningful interaction with the attacker but not as open as a High Interaction Honeypot (HIH). HIH is a computer with a real OS running vulnerable services to attract intruders to break into to capture their actions for analysis. Table 2 shows the pros and cons of the three types of honeypot.

**Table 2:** Honeypots type.

Honeypot type	Pros	Cons
LIH	Easy to manage, low risk for network	Easy to be noticed by the attackers
MIH	meaningful interaction with the attacker and allow the simulation of a service or operating system where everything is controlled	Need more network configuration to protect the honeypot network. It may endanger the network if the attacker fully controls it
HIH	The only type of Honeypot that provides bot binary information and execution code	High risk to the network operator requires more advanced configuration for the network and operations skills

**Figure 8:**
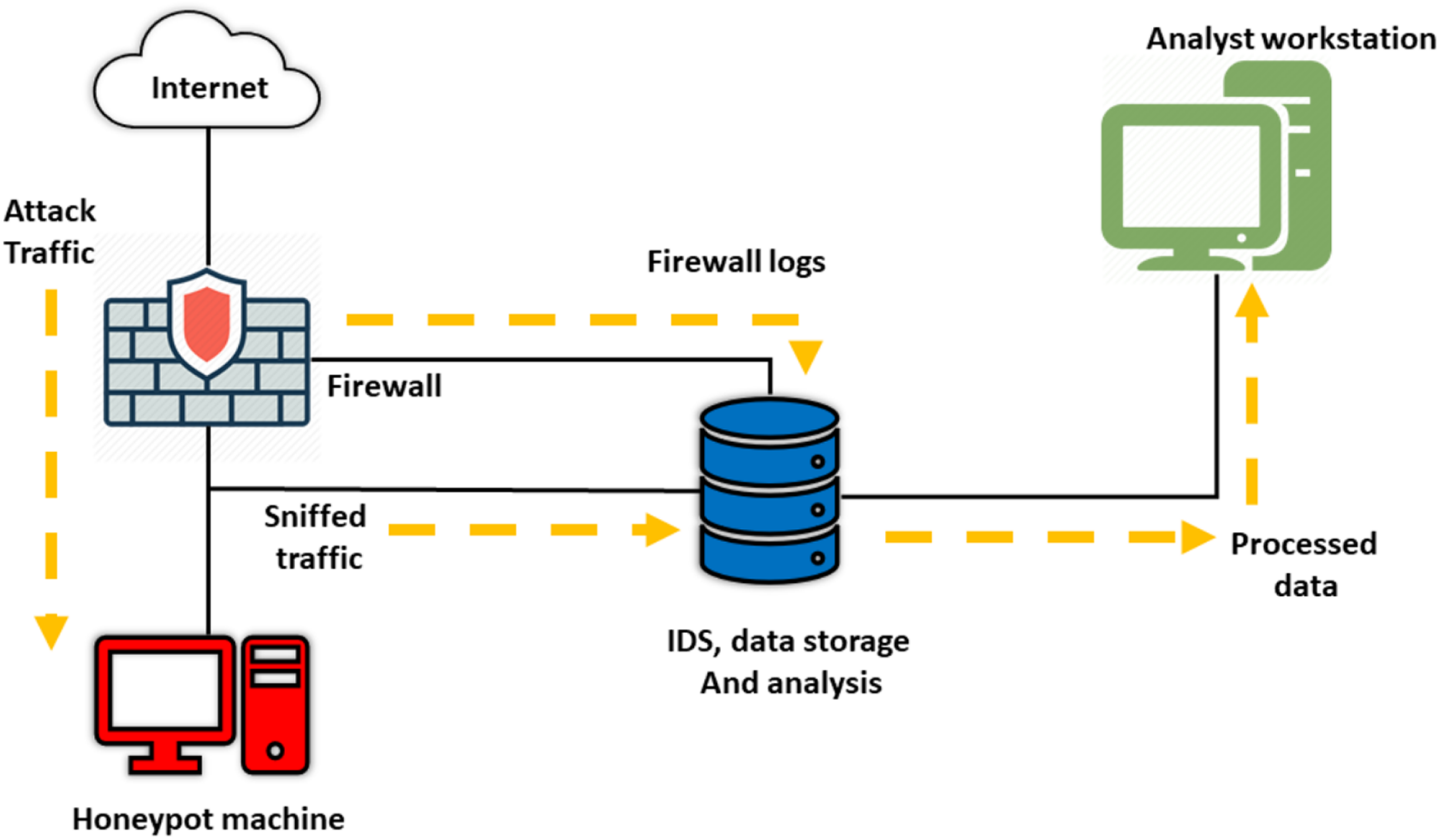
A typical Honeypot configuration.

Honeydns, proposed by Oberheide, Karir & Mao (2007), is a form of LIH that uses some simple statistics over the captured queries and collects DNS queries targeting unused (*i.e*., darknet) address spaces. This method prevents attackers from avoiding it (Bethencourt, Franklin & Vernon, 2005). However, a honeypot cannot detect all forms of bots, such as bots that are not using scanning to propagate (Dornseif, Holz & Klein, 2004). Furthermore, attackers could utilise honeypots to target other systems or machines outside the honeypots (Liu et al., 2009). Figure 8 shows the standard honeypot configuration.

Anirudh, Arul Thileeban & Nallathambi (2017) built a model using MIH as a sensor to collect attack logs. When coupled with an Intrusion Detection System (IDS) as a verifier, these logs increase 55–60% in IDS efficiency against DDoS attacks compared to using IDS alone. However, their research is limited to DDoS attacks only (Anirudh, Arul Thileeban & Nallathambi, 2017).

### Intrusion Detection System (IDS)

Da Luz (2014) and Alomari et al. (2016) categorised IDS into two: anomaly-based and signature-based (Da Luz, 2014; Alomari et al., 2016). The anomaly-based IDS can be further classified into host-based IDS and network-based IDS (Dornseif, Holz & Klein, 2004). The subsequent sections provide more details on the different types of IDS.

#### Signature-based Botnet detection

A signature-based detection method only detects botnets with matching predefined signatures in the database. DNS-based blacklist (DNSBL) method proposed by Ramachandran, Feamster & Dagon (2006) tracked DNS traffic and discovered bots’ identities based on the insight that botmasters could perform a “recognition” search to determine blacklisted bots. The limitations of the DNSBL-based approach are that it can only detect scouting botmaster and limited to bots propagated through SPAMs traffic using a heuristic approach.

#### Anomaly-based Botnet detection

Anomaly-based detection method relies on different DNS anomalies to identify botnets. Some of the DNS anomalies used for detection include high network latency, Time to Live (TTL) domain, patterns of domain requested per second, high traffic volumes, and irregular device behaviour that may expose bots’ existence. In other words, the term “detection based on anomaly” refers to the act of finding odd habits that differ from the expected ones. The anomaly-based approaches have two detection methods: host-based and network-based (Dornseif, Holz & Klein, 2004; Karim et al., 2014; Da Luz, 2014).

##### Host-based approaches

Host-based technique scans and protects the computing device locally, or in other terms, ‘host-level. Shin, Xu, and Gu proposed the EFFORT framework that combines several techniques to observe DNS traffic at the host level (Shin, Xu & Gu, 2012). EFFORT has five specific modules that use a controlled machine learning algorithm to report malicious domain names regardless of network topology or communication protocol and performs well with encrypted protocols. However, the EFFORT framework only worked with botnets that rely on the DNS administration to recognise C&C servers’ addresses. Host-based IDS is typically an inadaptable approach. Consequently, the observing agents must be deployed on all devices in the network to be effective against botnet attacks (Da Luz, 2014).

##### Network-based approaches

Network-based IDS analyses network traffic, either actively or passively (Dornseif, Holz & Klein, 2004; Karim et al., 2014; Da Luz, 2014). The active monitoring approach injects test packets into the network, servers, or applications instead of just monitoring or passively measuring network traffic activities.

**Active Monitoring Approaches**

Ma et al. (2015) proposed an active DNS probing approach to extensively determine unique DNS query properties from DNS cache logs (Ma et al., 2015). This technique could be used remotely to identify the infected host. However, injecting packets into the network increased the risk of revealing the existence of the IDS on the network. Furthermore, active analysis of DNS packets could threaten users’ privacy. Besides, the NXDOMAIN requests were absent from the DNS cache entry for domain names. The active monitoring mechanism added additional traffic from test and test packets injected into the network (Alieyan et al., 2016).

FluXOR (Passerini et al., 2008) is one of the earlier systems to detect and monitor fast-flux botnet. The detection technique is based on an interpretation of the measurable characteristics of typical users. It used an active sampling technique to track each suspected domain to detect the fast-flux domain. Not only can FluXORs recognise fast-flux domains, but also the number and identity of related proxy servers to prevent their reuse in a potential fast-flux service network (Monika Wielogorska, 2017). However, FluXOR is restricted to the fast-flux domains advertised by SPAM traffic (Perdisci, Corona & Giacinto, 2012).

**Passive Monitoring Approaches**

Passive monitoring utilises specific capturing instruments, known as “sensors,” to track the passing traffic. Subsequently, the traffic on the network under inspection would not increase. Weimer implemented the first passive detection method in 2005 (Weimer, 2005; Zdrnja, Brownlee & Wessels, 2007).

NOTOS (Antonakakis et al., 2010) is a comprehensive domain name reputation system that analyses DNS and secondary data from honeypots and malware detection services. Reputation process inputs are the characteristics derived from the list of domain names, such as the resolved IP address, the domain registration date, identified malware samples accessing a given domain name or IP address, and domain name blacklisted IP addresses. These features allowed NOTOS to change the domain legitimacy model, clarify how malicious domains are run, and calculate the perfect reputation score for new domains. NOTOS has high accuracy and low false-positive rate and can identify newly registered domains before being released on the public blacklist. However, a reputation score algorithm needs a domain registration history (whois), which is not available for all domain names, to award an appropriate reputation score. It is also unusable against frequently shifting C&C domains, such as a hybrid botnet that uses several C&C server hubs to execute commands (Kheir et al., 2014).

Contrary to NOTOS, Mentor (Kheir et al., 2014) proposed a machine learning approach on a statistical set of features. The proposed model sought to exclude all valid domains from the list of blacklisted C&C botnet domains, which helped to minimise both the false-positive rate and domain misclassification during the identification process. To do this, Mentor embedded a crawler to gather data on suspicious domain names, *e.g*., web content and domain properties, to create a DNS pruning model. The Mentor method’s performance is better when measured against public blacklist domains with meagre false-positive rates.

EXPOSURE is a system proposed by Bilge et al. (2011) that used inactive DNS information to identify domains vulnerable to malicious behaviour. It held a total of 15 features distributed over four classes: time-based, DNS-based, TTL-based, and domain-based. It also used these features to improve the training of PART classifiers.

Kopis introduced a new traffic characteristic by analysing DNS traffic at top-level domain hierarchy root levels (Antonakakis et al., 2011). This method reliably looked at the malware used domains by going through global DNS query resolution patterns. Unlike other DNS reputation strategies such as NOTOS and EXPOSURE, Kopis allowed DNS administrators to freely inspect malware domains without accessing other networks’ data. In addition, Kopis could search malware domains without access to IP reputation info (Xu et al., 2017).

Pleiades (Antonakakis & Perdisci, 2012) helped classify recently controlled DGA domains using non-existent domain responses (NXDOMAIN). However, because its clustering strategy relied on domain names’ structural and lexical features, it was limited to DGA-based C&C only. Also, one of the outstanding issues of NXDOMAIN-based detection was dealing with a compromised host with malware that requested several queries to DGA domains over an extended time. It might be possible to detect the C&C addresses of a domain fluxing botnet in the local network by comparing the accurate domain resolution entropy to the missed one (Yadav et al., 2010). Since the randomness in the domain name alphanumeric characters is measurable by calculating the entropy value, in their implementation, the researchers utilised an offline IPv4 dataset from the Asian region. They achieved a low FP rate of just 0.02%. However, their approach was limited to non-dictionary IPv4 domain names.

There has been extensive discussion on botnet detection approaches that employ machine learning detection in the literature. For example, BOTCAP (Gadelrab et al., 2018) utilises J48 and ‘Support Vector Machine’ (SVM) classifiers for training the extracted DNS features. The authors showed that the J48 classifier, a Java version of the C4.5 classifier, performed better than other classifiers. However, a hybrid detection model that combines the output of the J48 classifier with other classifier models’ output could further improve the performance.

Li et al. (2019) attempted to find the best classifiers from several classifiers, such as Decision Tree-J48, ‘Artificial Neural Network’ (ANN), ‘Support Vector Machine’ (SVM), Logistic Regression, ‘Naive Bayes’ (NB), ‘Gradient Boosting Tree’ (GBT), and ‘Random Forest’ (RF) (Li et al., 2019). As a result, the authors showed that J48 was the best classification algorithm to classify the DGA domain (Li et al., 2019). However, their proposed approach was not using any hybrid rule model.

Haddadi et al. (2014) adopted the C4.5 classifier for botnet classification (Haddadi et al., 2014). However, the selected subset of features did not contribute to any improvement in the classification process. The experimental results achieved an 87% detection rate.

Likewise, deep learning, a subset of machine learning, has received significant attention lately. A deep learning algorithm of recurrent neural networks (RNN), long short-term memory (LSTM), and the combination of RNN and LSTM have been applied as a botnet detection method (Shi & Sun, 2020). The RNN and LSTM combination achieved higher detection results. However, deep learning techniques require massive pre-processing of data, long process time, and resources with high-speed processors. Besides, to discover new bots, re-training the whole model with a new dataset is a must. Re-training is a time-consuming process and not suitable for detecting new botnets.

From the literature above, it is noticeable that there is a lack of significant features and rules that contribute to detecting DNS-based botnet with high accuracy and low false-positive rate.

The summary of some existing botnet detection approaches based on DNS traffic analysis are tabulated in Table 3.

**Table 3:**
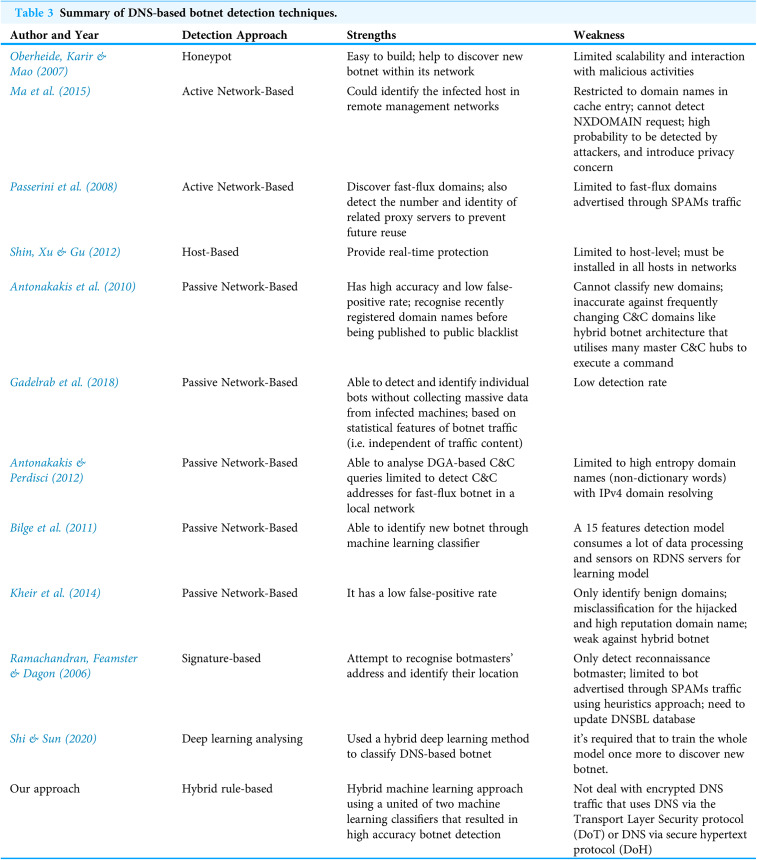
Summary of DNS-based botnet detection techniques.

Author and Year	Detection Approach	Strengths	Weakness
Oberheide, Karir & Mao (2007)	Honeypot	Easy to build; help to discover new botnet within its network	Limited scalability and interaction with malicious activities
Ma et al. (2015)	Active Network-Based	Could identify the infected host in remote management networks	Restricted to domain names in cache entry; cannot detect NXDOMAIN request; high probability to be detected by attackers, and introduce privacy concern
Passerini et al. (2008)	Active Network-Based	Discover fast-flux domains; also detect the number and identity of related proxy servers to prevent future reuse	Limited to fast-flux domains advertised through SPAMs traffic
Shin, Xu & Gu (2012)	Host-Based	Provide real-time protection	Limited to host-level; must be installed in all hosts in networks
Antonakakis et al. (2010)	Passive Network-Based	Has high accuracy and low false-positive rate; recognise recently registered domain names before being published to public blacklist	Cannot classify new domains; inaccurate against frequently changing C&C domains like hybrid botnet architecture that utilises many master C&C hubs to execute a command
Gadelrab et al. (2018)	Passive Network-Based	Able to detect and identify individual bots without collecting massive data from infected machines; based on statistical features of botnet traffic (i.e. independent of traffic content)	Low detection rate
Antonakakis &Perdisci (2012)	Passive Network-Based	Able to analyse DGA-based C&C queries limited to detect C&C addresses for fast-flux botnet in a local network	Limited to high entropy domain names (non-dictionary words) with IPv4 domain resolving
Bilge et al. (2011)	Passive Network-Based	Able to identify new botnet through machine learning classifier	A 15 features detection model consumes a lot of data processing and sensors on RDNS servers for learning model
Kheir et al. (2014)	Passive Network-Based	It has a low false-positive rate	Only identify benign domains; misclassification for the hijacked and high reputation domain name; weak against hybrid botnet
Ramachandran, Feamster & Dagon (2006)	Signature-based	Attempt to recognise botmasters’ address and identify their location	Only detect reconnaissance botmaster; limited to bot advertised through SPAMs traffic using heuristics approach; need to update DNSBL database
Shi & Sun (2020)	Deep learning analysing	Used a hybrid deep learning method to classify DNS-based botnet	it’s required that to train the whole model once more to discover new botnet.
Our approach	Hybrid rule-based	Hybrid machine learning approach using a united of two machine learning classifiers that resulted in high accuracy botnet detection	Not deal with encrypted DNS traffic that uses DNS via the Transport Layer Security protocol (DoT) or DNS via secure hypertext protocol (DoH)

## Materials & methods

**Figure 9:**
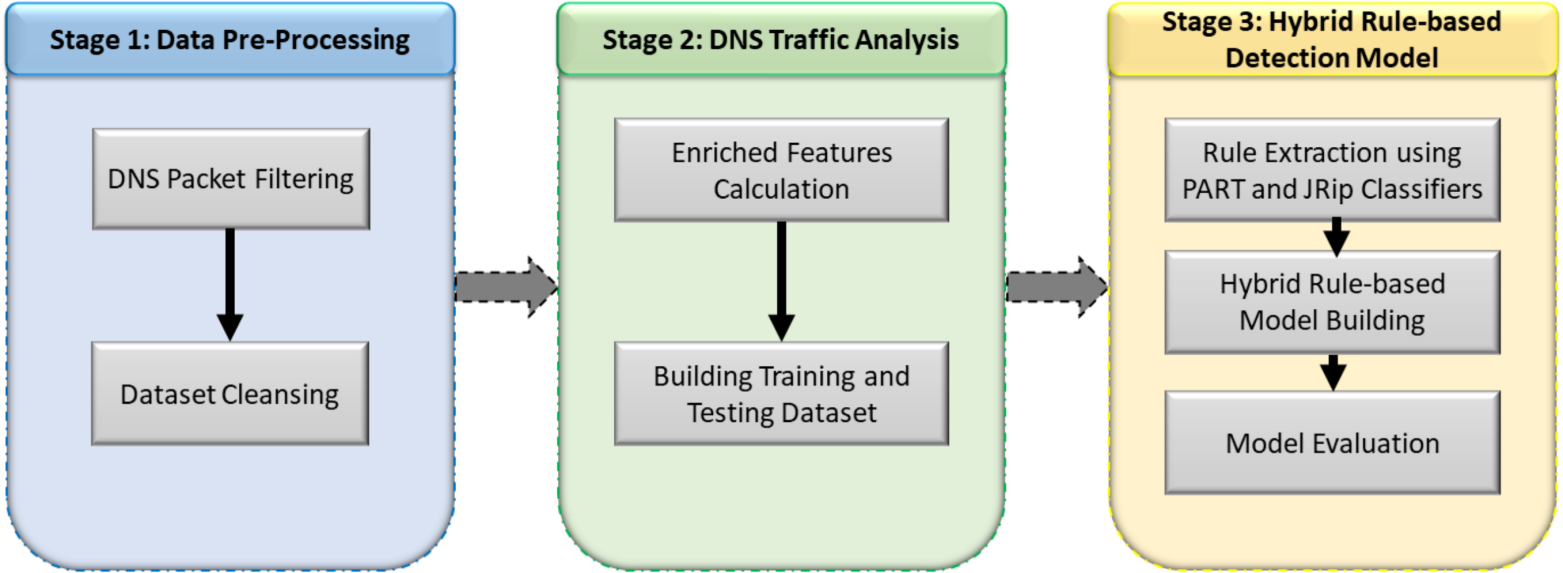
Three stages of the detection method design.

This section thoroughly explains the materials and methods used to implement the proposed approach. The proposed approached consists of three stages, as shown in Fig. 9.

The following subsections provide complete detail of each stage.

### Stage 1: data pre-processing

Data pre-processing stage is critical for the proposed approach. It helps to focus on the required DNS features to provide a more flexible selection analysis. Also, this process reduces the analysis time and false-positive results as well.

It consists of two steps, DNS packet filtering and data cleansing. The packet filtering step ensures that only DNS packets remain in the filtered network traffic. Furthermore, this research assumes that a third-party security mechanism is deployed in the network to prevent or detect DNS fragmentation packets. Therefore, the proposed approach incorporates the third-party mechanism to ensure that the DNS fragmented packet will not bypass the proposed rules.

### DNS packet filtering step

**Figure 10:**
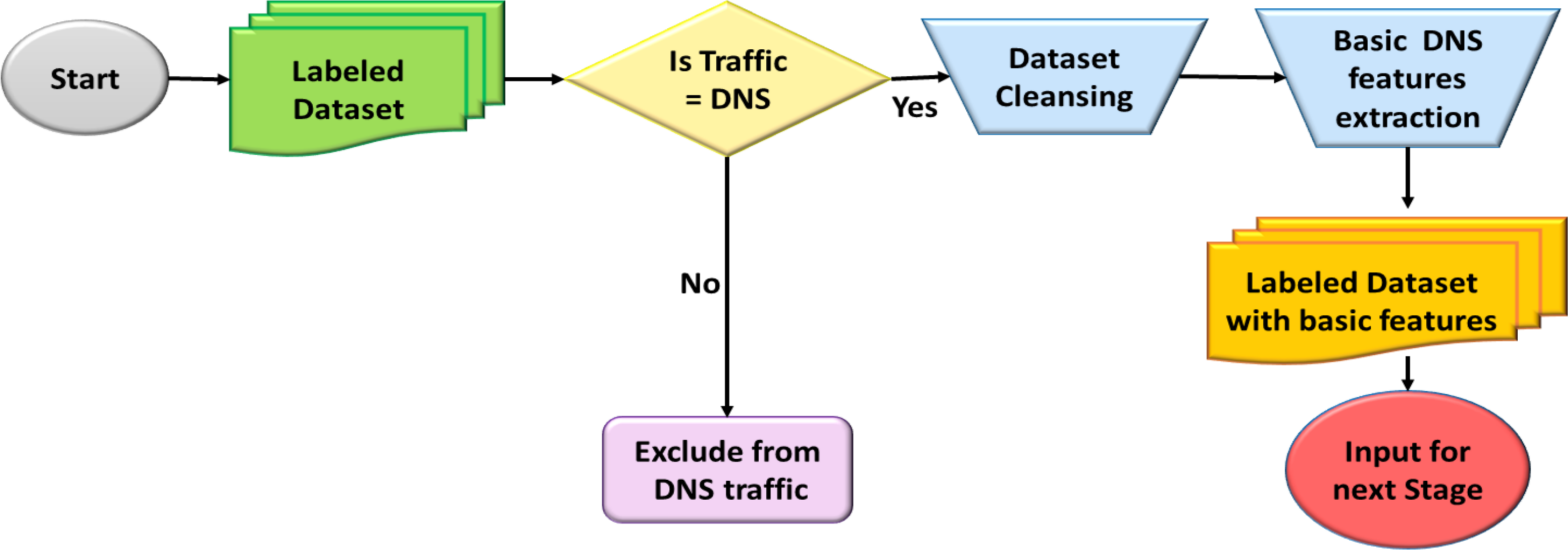
Flowchart for data pre-processing stage.

The process of resolving DNS queries occurs nearly instantaneously most of the time. Since there is no need for a handshaking technique provided by Transmission Control Protocol (TCP), DNS traffic uses User Datagram Protocol (UDP) at port 53, making the filtering process easier. Furthermore, this study focuses on the analysis of selected features of DNS. The filtering step is responsible for the extraction of the required DNS features from DNS packets. Figure 10 illustrates the process of the data pre-processing stage.

**Figure 11:**
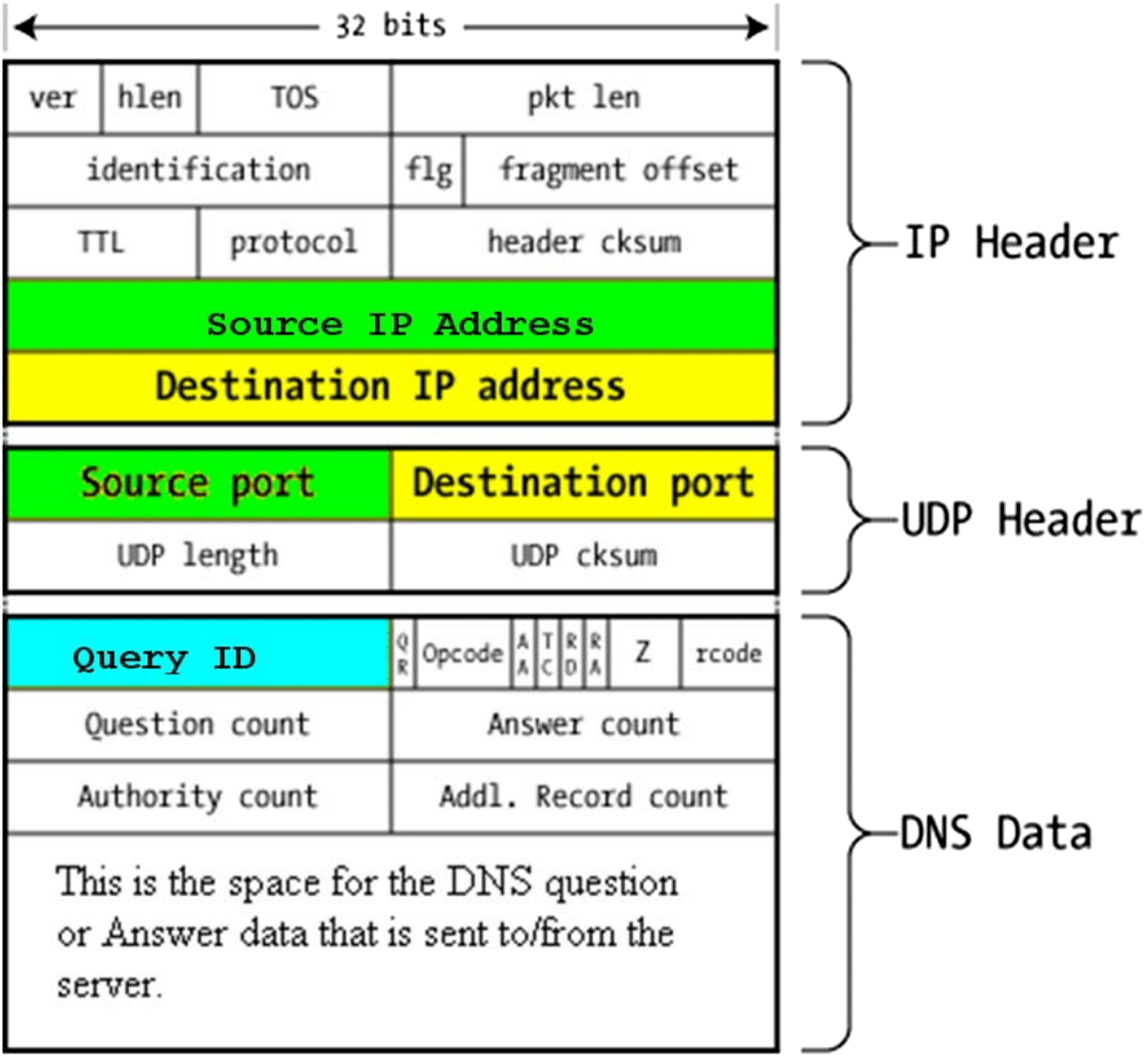
DNS packet structure.

****Figure 11 visualises the DNS packet structure. Table 4 tabulates the extracted DNS traffic fields selected for this study. Finally, Table 5 presents the extracted DNS record types with their function in the DNS protocol.

**Table 4:** Extracted DNS traffic basic features.

Fields	Description
TIME	Traffic time
Source IP address	Sender (host) IP address
Destination IP address	Receiver (host) IP address
QR (Query/response)	A one-bit field that specifies whether this message is a query (0), or a response (1).
RCODE	4-bit field is set as part of responses with these values: 0 No error 1 Format error 2 Server failure 3 Name Error 4 Not Implemented
QNAME	Domain name requested
TTL (DNS response)	Time to Live (TTL) of Resource Record (RR). A 32-bit integer in seconds, primarily used by resolvers when they cache RRs. Describes how long to cache RR before discarded.

**Table 5:** DNS record types.

DNS record type	Description	Function and implication
A	IPv4 address record	A 32-bit IP Host address. A connection to this IP address by the user will follow
AAAA	IPv6 address record	A 128-bit IP Host address. A connection to this IPv6 address of the user will follow
CNAME	Canonical name record	Mapping domain name to another domain DNS query with the value of the CNAME from the response as the QNAME of the query might follow
MX	Mail exchange record	Maps a domain name to mail server agent. A mail transfer to this server might follow
NS	Name server record	Delegates a DNS zone to name servers. Implication: DNS queries to these servers might follow.
PTR	Pointer record	Used in reverse DNS lookups
SIG	Signature	Signature record
SOA	Start of authority record	Provide valuable information about the domain, including the primary name server, administrator email, the serial number and TTL
SRV	Service Locator	Generalised service location record used for newer protocols instead of creating protocol-specific records such as MX. Inference: A connection to the A record of the hostname with the specific parameters might follow. Compared to the A record alone, an observer of a query for an SRV record knows precisely what type of connection to the IP address of the hostname might follow.
TXT	Text record	Used to carry text data. Text data could be readable, or machine-generated text.

### Data cleansing step

Cleansing the data means removing errors and broken DNS sessions from the datasets. Thus, the cleaning process helps achieve more accurate results and reduces the processing time of subsequent stages (Alieyan et al., 2021).

### DNS traffic analysis

The DNS traffic analysis stage consists of enriched features calculations (feature engineering) and building training dataset steps. The following subsection provides a more detailed explanation for each step.

### Enriched features calculations (feature engineering) step

The feature engineering process employs different machine learning domains to solve various types of problems. Its main task is to select and compute the most significant features or attributes and eliminate irrelevant and redundant features to improve machine learning algorithms’ performance. In this study, the feature engineering process derives enriched DNS features from the basic extracted features in Stage 1.

Based on the review of existing literature and studies, we considered two significant characteristics of DNS-based botnet in its connection phase. Firstly, DNS-based botnet generates a massive number of domain names. Secondly, the generated domain names tend to be random and different from the human-generated ones (Alieyan et al., 2021).

The calculation of randomness of domain names could help to distinguish anomalous traffic and benign traffic. In information theory, the randomness could be calculated by the Shannon entropy equation, first introduced by Claude E. Shannon in his paper titled “A Mathematical Theory of Communication” (1948). Shannon entropy allows estimating “the average minimum number of bits needed to encode a string of symbols based on the alphabet size and the frequency of the symbols.” Moreover, Shannon entropy is also being applied in information and network analysis. Therefore, the proposed approach employs the Shannon entropy algorithm to calculate the resolved domain name’s entropy, using Eq. (1).

(1) $$H\left( x \right) = \mathop \sum \limits_{i = 1}^n p\left( {{x_i}} \right)I\left( {{X_i}} \right) = \mathop \sum \limits_{i = 1}^n p\left( {{x_i}} \right)\log {\rm \; }\displaystyle{1 \over {p\left( {{x_i}} \right)}} = - \mathop \sum \limits_{i = 1}^n p\left( {{x_i}} \right)\log {\rm \; }p\left( {{x_i}} \right)$$

Since bots repeatedly tried to connect with the botmaster’s C&C server, the number of domain resolution requests will be high. The proposed methodology for traffic analysis is to group the requested domain according to source IP. Since the bot or botnet tries to connect with the botmaster in different predefined periods, the average entropy for the source IP is essential to distinguish between benign and malicious traffic. Furthermore, we use the same time value, 5 s, for flow analysis based on a previous study (Alieyan, 2018). Equation (2) calculates the average domain entropy feature (F1).

(2) $$\overline {H\left( x \right)} = \displaystyle{{\mathop \sum \nolimits_{i = 1}^N {H_{\left( {xi} \right)}}} \over N}$$

where N denotes the number of domain requests in a predefined time (5 s), and ${H_{\left( x \right)}}$ is as mentioned in Eq. (1). Moreover, as previously mentioned, a botnet in the rallying phase repeatedly tries to connect with its C&C server. Since the C&C server is usually configured with a single or only a few domains from the pool of vast numbers of bot-generated domain names, many failed domain name resolution requests occur before the bot successfully connects with the registered C&C domains. Such actions will increase the NXDOMAIN response ratio from the infected network or host, indicating anomalous behaviour (Wang et al., 2017). Furthermore, regular users usually have different domain request time patterns, whereas the infected host endeavour to connect with their C&C server according to a pre-programmed schema. Consequently, the time for domain request entropy in legitimate hosts diverges from the infected ones (Qi et al., 2018).

Furthermore, the values of legitimate DNS lookup type requests and DNS record types, as stated in Table 5, will differ from the values in an infected host since that user’s behaviour in requesting domain resolution is different from the bot-generated request (Hikaru et al., 2018). Likewise, the attackers exploit fast-flux by combining round-robin IP addresses with a short TTL for the DNS Resource Record (RR) (William & Danford, 2008), leading to different TTL settings for the malicious domains.

**Table 6:** The resulted subset of features in the training dataset.

F#	Feature Name	Description
1	Avg_domain_ent	Average requested domains entropy at a predefined-time.
2	No_suc_resp	The total number of successful responses in predefined-time.
3	rand_query	The randomness of the number of DNS queries rate in the predefined-time interval.
4	number_of _record type	The number of records requested in a predefined-time.
5	Avg_TTL	Average Time to Live in a predefined-time, TTL defines how long the response record for a domain should be cached in the DNS server or the host.
6	No_Distinct_TTL	The total number of different values for TTL values in the predefined-time.
7	No_Distinct_Packet	The total number of different sizes of packets in predefined-time.
8	No_Distinct_Destination	The total number of different destinations in predefined-time.
9	No_error_resp	The total number of unsuccessful (error) responses in predefined-time.
10	Ratio_suc_resp	The ratio of successful response in a predefined-time.

Based on the characteristics mentioned above, the equations for the calculation of the enriched feature are as follows:

*R* is the ratio of the successful DNS response within a predefined time, which is also the definition of the second feature (F2):

(3) $$R = \displaystyle{{{R_s}} \over {{R_n}}}$$

where ${R_s}$ represents the number of successful DNS responses, and ${R_n}$ represents the number of DNS requests.

*H(q)* is the randomness number of DNS queries rate within a predefined time interval. It is calculated according to the Shannon entropy stated in Eq. (1). Thus, the definition of the third feature (F3) is calculated by:

(4) $$H\left( q \right) = - \mathop \sum \limits_{x = 1}^N {\rm \; }\left( {\displaystyle{{{q_x}} \over {\mathop \sum \nolimits_{x = 1}^N {q_x}}}log\displaystyle{{{q_x}} \over {\mathop \sum \nolimits_{x = 1}^N {q_x}}}} \right)$$

where ${q_x}$ represents the number of DNS queries in an $x$ time interval, and *N* refers to the total number of DNS queries type (Qi et al., 2018).

${\rm D}\Delta t$ is the number of resolved DNS record types within a predefined time interval. The definition of the fourth feature (F4) is as follows:

(5) $${\rm D}\Delta t = \mathop \sum \limits_{i = 1}^N \left( {{D_i}} \right)$$

where $\Delta t$ represents the predefined time, ${D_i}$ represents the number of the *i-*th DNS request type as tabulated in Table 5, and *N* denotes the total number of DNS requested.

The average of the resolved domain name TTL in a predefined time interval, which is the definition of the fifth feature (F5), is measured by:

(6) $$TT{L_{\mu {\rm \; }}} = \displaystyle{{\mathop \sum \nolimits_{i = 1}^N TT{L_i}} \over N}$$

The total number of various values for TTL within a predefined-time (F6).

The total number of different sizes of DNS packets within a predefined-time (F7).

The number of different DNS destinations within a predefined-time (F8).

The total number of unsuccessful (error) DNS response within a predefined-time (F9).

The ratio of successful DNS response in a predefined-time (F10).

### Building training dataset step

The objective of this step is to construct a training dataset to train the machine learning classifiers. The training dataset comprises a set of enriched features computed through a feature engineering process. As mentioned earlier, the features are calculated based on 5 s running time series of the source IP that resulted in a network traffic flow defined as unidirectional traffic with certain packet features that represent a flow tuple (Krmicek, 2011). In this study, the features that describe the flow are the source IP, destination IP (DNS server), and protocol (DNS). Furthermore, the total number of domain requests is one of the features available in the flow but not in the individual packet (Haddadi & Zincir-Heywood, 2015). The use of traffic flow helps to reduce both the training time and the number of process instances. Even though the per-packet analysis is accurate, it requires extensive resources and cannot efficiently deal with encrypted network traffic (Zhao et al., 2013).

Additionally, to avoid being misled while building the rule model, the rule extraction process will remove the source IP address feature used for flow creation since the source IP address in the actual traffic might differ from data collection traffic.

Furthermore, the dataset is presented as a grouped aggregated flow. For a unified grouped aggregated flow time during the calculation of the computed features, the predefined time used for each calculated group is 5 s based on previous studies (Alieyan, 2018; Qi et al., 2018). Additionally, by aggregating the flow in a fixed interval of 5 s, the dataset size and the processing time are reduced. Table 6 tabulates the extracted set of basic features with enriched features.

### Stage 3: hybrid rule-based detection model

This stage presents a hybrid rule-based detection model to detect botnet attacks in DNS traffic. The hybrid-rule model is built using the PART and JRip machine learning algorithms. To properly assess the proposed approach’s performance, a ten-fold cross-validation method (Kohavi, 1995) is utilised to select the best model for rule detection.

The PART classification algorithm is a Java-based variation of the C4.5 algorithm (Salzberg, 1994; Thankachan, 2013) and different SVM kernels (Hsu, Chang & Lin, 2003; Chang & Lin, 2011). C4.5 is a popular decision tree supervised classifier widely used in data mining. The C4.5 decision tree is generated based on the provided classes and feature sets (Alazab et al., 2011).

JRip (Repeated Incremental Pruning) is the Weka variant of Repeated Incremental Pruning to Produce Error Reduction (RIPPER), suggested by William W. Cohen as an enhanced version of IREP (Hall et al., 2009). JRip offers a range of capabilities that could improve detection accuracy, such as a technique to revise and replace generated rules, deal with noisy data, and fix over-the-counter issues. In addition, JRip optimises the rule set by the re-learning stage, leading to higher accuracy as the rules are regularly revised. Its classifier performs well even for imbalanced class distribution (Hall & Joshi, 2005; Qazi & Raza, 2012; Napierala & Stefanowski, 2016).

**Figure 12:**
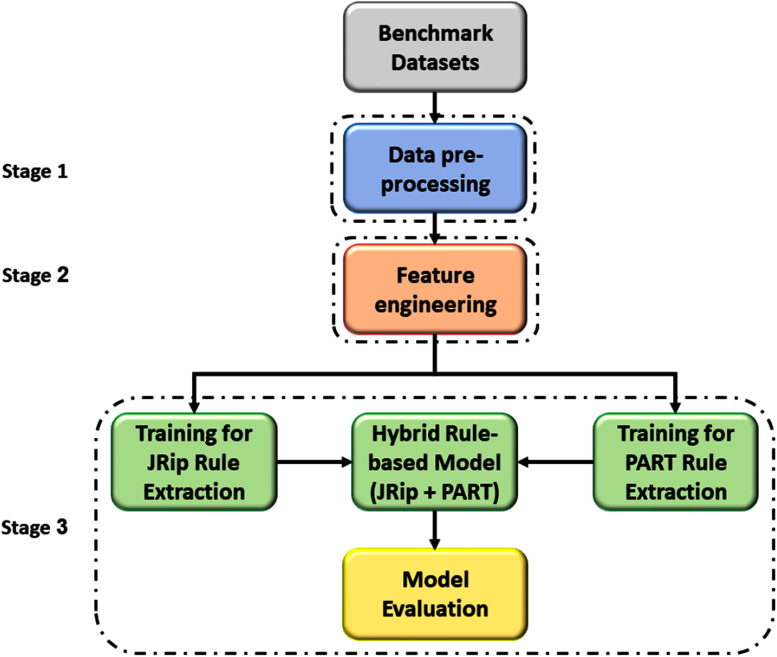
Process of a proposed hybrid rule-based model.

In this study, we selected PART and JRip machine learning classifiers for several reasons. Firstly, JRip and PART are sets of non-complex rules and could be integrated easily with any IDS system. Secondly, even though other classification algorithms are available, JRip and PART classifiers are used by many researchers in their recent work (Faizal et al., 2018; Kumar, Viinikainen & Hamalainen, 2018; Adewole et al., 2019). Thirdly, the proposed approach assumed that the hybridisation of the two classifiers would improve the output result; thereby, the final detection model rule is a hybrid of extracted rules from both PART and JRip output. Both JRip and PART classifiers require a training dataset. The extracted model for each classifier output, including the hybrid set of rules, is evaluated using 10-fold cross-validation. Figure 12 illustrates the process of the proposed hybrid rule-based model for the detection of DNS-based botnets.

### Implementation environment

The software used includes Microsoft’s Windows 10 (64-bit) operating system, WEKA version 3.8, Microolap TCPDUMP for Windows® 4.9.2, Wireshark 3.02, and Python 2.8.

We also utilised the WEKA tool to extract the detection rules using the built-in JRip and PART algorithms. It is a set of machine learning algorithms for different data-mining tasks, such as data pre-processing, classification, and clustering.

In addition, Microolap TCPDUMP for Windows®, a network traffic sniffer and analyser software, was used to extract DNS traffic from the benchmark dataset. Wireshark is a network protocol analyser tool used for detailed analysis and basic feature extraction of DNS packets. We used a python script in conjunction with Wireshark to calculate the new enriched features. The results of feature extraction, tabulated in Table 6, were stored in a comma-separated values (CSV) file. Furthermore, having the final training file in CSV file format ensures seamless compatibility since it is fully supported and readable by WEKA.

Finally, The hardware used in this study consists of a CPU with an Intel® Core™ i5-8250u processor, 8 GB of memory, and a 256 GB Solid State Drive (SSD) hard disk.

### Benchmark datasets

The experiment of this research is validated using two benchmark datasets: Network Information Management and Security Group (NIMS) dataset (Haddadi & Zincir-Heywood, 2016) and CTU13 dataset (Garcia et al., 2014).

The NIMS dataset by the Network Information Management and Security Group of Dalhousie University in Halifax, Nova Scotia, Canada, contains four distinct traces: a normal traffic trace based on Alexa domain ranks and three different traces of malicious traffic from Citadel, Zeus, and Conficker botnets. Table 7 lists the number of domain names inside the dataset for each trace.

**Table 7:** NIMS dataset domains count.

Dataset	Record count	Size (MB)
Alexa (normal traffic)	654	2.2
Citadel	1,331	9
Zeus	707	11
Conficker	98,606	1,800

The experiment in this study utilised the regular DNS traffic data within the CTU13 dataset (“Index of/publicDatasets/CTU-Normal-4-only-DNS,” 2016; https://mcfp.felk.cvut.cz/publicDatasets/CTU-Normal-4-only-DNS/). The CTU13 dataset contains 5,966 normal DNS traffic packets. The dataset comprises traffic collected from music streaming service 20songstogo.com, Gmail, Twitter, and regular web surfing *via* the Google Chrome browser.

Recently, many researchers used the CTU13 dataset in their work (Haddadi, Phan & Zincir-Heywood, 2016; Chen et al., 2017; Pektaş & Acarman, 2019).

The non-malware traffic used in this experiment is from the normal part of CTU13, which is CTU4 and CTU6 (“Malware Capture Facility Project: Normal Captures—Stratosphere IPS”; https://www.stratosphereips.org/datasets-normal). The normal traffic for CTU4 is from a home computer network and includes only regular DNS traffic for privacy reasons. Similar to CTU4, the CTU6 comprises regular DNS traffic generated from a Linux-based notebook in a university network.

**Figure 13:**
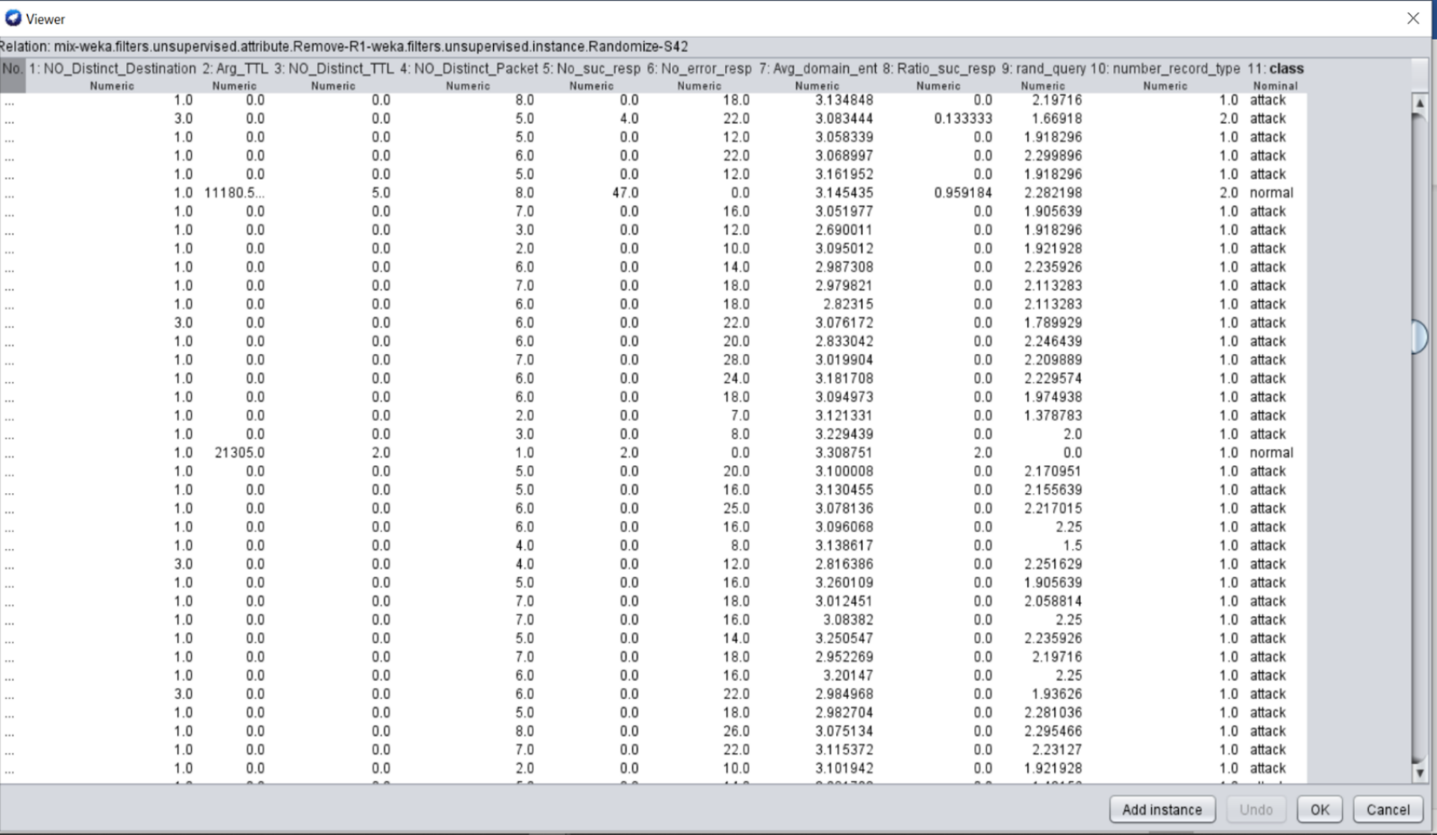
Snapshot of training dataset instances.

Finally, for our static analysis purpose, two enriched datasets were extracted using feature engineering. The first dataset is a mixed dataset that combines both NIMS and CTU13 (normal traffic) datasets, and the second dataset is based only on NIMS datasets. The combination of normal traffic is to reduce overfitting resulted from an imbalance class. Figure 13 shows a sample snapshot of training dataset instances.

It can be noticed that the datasets used for evaluating our proposed approach were from 2014 and 2016. However, using these datasets will not impact the presented result for the following reasons: (i) in our approach, we analysed botnet’s DNS communication patterns, which are totally different from human DNS communication. There is no newer dataset publicly available that fulfils our requirement (DNS-based botnet traffic), and (ii) these datasets were also used by other researchers in their works (as recent as 2020) that we are comparing with. Therefore, we also need to benchmark our proposed work using the same dataset for fair evaluation and comparison.

Furthermore, our proposed work relies on the core DNS features that will always exist in the DNS-based botnet lifecycle, which remains the same as long as it uses the conventional DNS protocol. Therefore, the use of these datasets should not render our proposed approach ineffective in detecting novel or future DNS-based botnets.

### Design of the proposed technique

The design of the proposed technique, illustrated in Fig. 9, consists of three stages. This section describes the design of each stage.

### Design of pre-processing stage

In this stage, first, the TCPDUMP tool selected and filtered DNS traffic from the network traffic, which reduced network traffic by 68%. This process will reduce the time and resources needed to analyse the remaining traffic. Then, several Wireshark DNS packet filters are used to extract several basic features from the DNS traffic.

Table 8 shows the extracted features and the corresponding Wireshark filters used. The basic extracted DNS features are stored in a CSV file as input for the next stage.

**Table 8:** List of extracted features using Wireshark filters.

Feature name	Feature description	Type of Wireshark filter
Time	The time a packet is captured	UTC date, as YYYY-MM-DD and time
Source IP address	The IP address for sender machine	DNS and ip.src
IP-TTL (Time To Live)	The time interval for cache before expiring for IP address	ip.ttl
Query ID	A 16-bit unique identifier assigned by the program that generates any query; allows the server to associate the answer with the question (query).	DNS.id
QR (Query/Response)	A one-bit field that specifies whether this message is a query (0), or a response (1).	dns.flags.response == 0 (query) dns.flags.response == 1 (response)
RCODE	This 4-bit field is set as part of responses with these values: No error Format error Server failure Name Error Not Implemented	dns.flags.rcode
QNAME	A domain name represented as sequence of labels, where each label consists of a length octet followed by that number of octets	dns.qry.name
TTL (DNS Response)	Time to Live (TTL) of the Resource Record (RR); a 32-bit integer in seconds; primarily used by resolvers when caching RRs; describes how long to cache RR before discarded.	dns.resp.ttl
QTYPE	A two-octet code which specifies type of query; The values include all codes valid for a TYPE field, together with more general codes which can match more than one type of RR; used in resource records to distinguish types such as A, AAAA, NS, CNAME.	**dns.qry.type** dns.qry.type == 1 *A – IPv4 for Host Address* dns.aaaa *AAAA -- IPv6 Address* dns.cname *Canonical Name Record type* dns.ns *Name Server Record type* dns.mx.mail_exchange *Mail Exchange Record type*

### Design of DNS traffic analysis stage

In this stage, the enriched features are calculated based on the basic extracted DNS features from the previous stage. The datasets had been prepared and normalised to calculate the features as tabulated in Table 6.

The first feature is the average randomness in queried domain names (F1), calculated using Shannon entropy, and as described in Section “Materials & Methods”, the queried domains are aggregated according to the source IP address (src_IP) every 5 s. Then, a python script is used to compute the enriched features, including the average entropy (avg_domain_ent) as per Eq. (2).

To calculate the second enriched feature (F2), several Wireshark filters are used in the process. The successful response (dns.sec.resp) is extracted using (dns.flags.rcode == 0) filter; the number of DNS requests (dns.req.num) is extracted using the (dns.flags.response == 0) filter; and both (dns.sec.resp) and (dns.req.num) are aggregated for each 5-second period using (src_IP). The ratio of successful response is calculated using Eq. (3) where the aggregated successful response is divided by the aggregated number of requests.

For the third enriched feature (F3), the DNS query packet is extracted using (dns.flags.response == 0) filter every 5 s. The entropy of the DNS query is calculated using Eq. (4). For the fourth enriched feature (F4), the resolved DNS records number is extracted using (dns.qry) filter. The result is calculated every 5 s using Eq. (5).

For the fifth feature (F5), the value of TTL response is extracted using (dns.resp.ttl) filter; then, the average response TTL is calculated using Eq. (6).

The rest of the features from F6 to F10 are calculated by following the same methods of using Wireshark filters, as shown in Table 8. The calculated DNS features are prepared as input for the next stage and stored in a CSV file. It is then considered as a labelled training dataset with only new DNS features. Table 9 shows the final number of dataset records after performing the flow aggregation.

**Table 9:** Total number of dataset instances.

Dataset	Instances	
	Attack	Normal
NIMS-based dataset	44,577	100
Mixed dataset	44,577	625

### Design of rule-based detection stage

**Figure 14:**
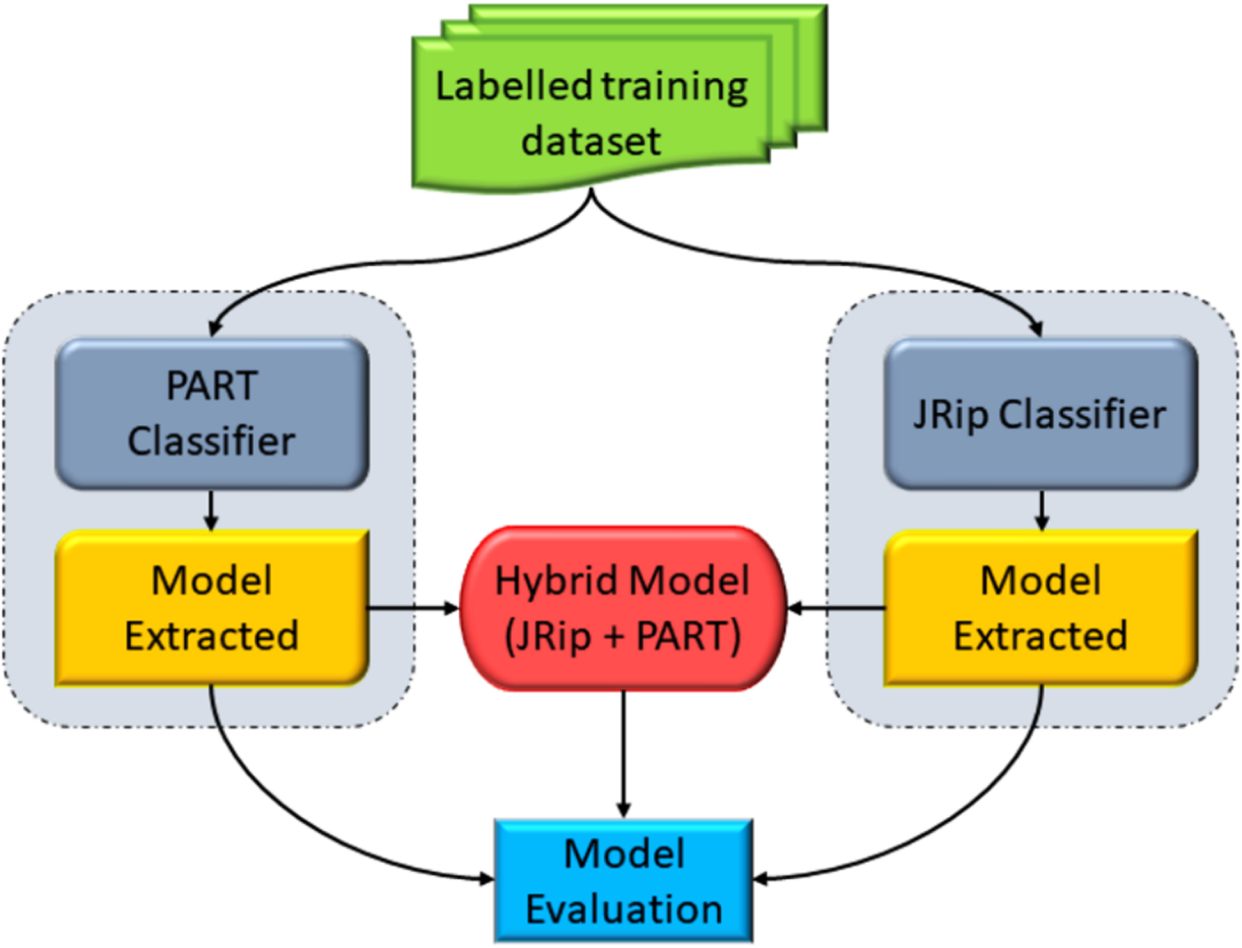
Rules extraction process.

In this stage, the Weka tool is used to extract botnet-based DNS detection ruleset using both PART and JRip classifiers. Initially, the enriched training dataset is the input for both PART and JRip classifiers. Then, to properly assess the predictive performance and overcome any bias in this process, the k-fold cross-validation training technique is used with the value of k set to 10 to build and test the model (Luo et al., 2017). Figure 14 illustrates the rules extraction process in this stage. Appendices A1, A2 and A3, provide in details the extracted rules for each used classifier.

## Results

**Figure 15:**
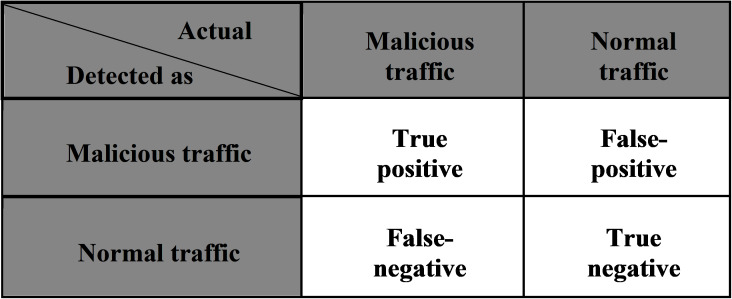
Evaluation metrics.

The three extracted models are evaluated using two different benchmark datasets (NIMS and CTU13) to measure the detection accuracy and false-positive rate, as shown in Eqs. (7)–(10). These evaluation metrics are computed by the parameters of the confusion matrix, as stated in Fig. 15. Many researchers adopted these evaluation metrics in their work (Soltanaghaei & Kharrazi, 2015; Kwon et al., 2016; Alieyan, 2018; Shi & Sun, 2020).

(7) $$Detection\; accuracy = \displaystyle{{TP + TN} \over {TP + TN + FP + FN}}$$

(8) $$False\; Positives\; rate = \displaystyle{{FP} \over {FP + TN}}$$

(9) $$Precision = \displaystyle{{TP} \over {TP + FN}}$$

(10) $$F1\; score = \displaystyle{{2TP} \over {\left( {2TP + FP + FN} \right)}}$$

Precision (proportion of correctly reported anomalies) and Recall (share of correctly reported anomalies compared to the total number of anomalies), Recall is another option which calculated implicitly using the F-measure. F-measure (F1) is a function that represents the relationship between Precision and Recall; a higher F-measure indicates a more accurate classification output.

Furthermore, to select the best detection model for the DNS-based botnet detection approach, the extracted rules for each classifier are separately evaluated using the cross-validation technique. The model with the highest detection accuracy was selected. The cross-validation experiments were conducted using a mixed dataset and (NIMS) dataset. Table 10 presents the result of the extracted rules and models and model complexity for each dataset.

**Table 10:** The results of the proposed approach.

Datsaets	Algorithms	Accuracy%	Precision	F1 score	FP rate%	Time Complexity (sec)	Rules complexity
MIXED	JRip	99.87	99.94	99.931	4.34	5.23	10
	PART	99.9	99.95	99.949	3.84	0.8	22
	Hybrid (JRip+PART)	99.96	99.97	99.977	1.6	6.03	32
NIMS	JRip	99.94	99.97	99.967	13	1.34	5
	PART	99.95	99.97	99.974	11	0.66	10
	Hybrid (JRip+PART)	99.97	99.98	99.988	5	2	32

Model complexity can be measured using various criteria, including memory consumption, time, and the number of the detection rules extracted using learning algorithms. Two complexity criteria are used in this work: (i) the estimated training time, which depends on the research platform, and (ii) the complexity of the model based on the number of extracted detection rules.

We can notice from Table 10 that the maximum time required to build the final model is 6.03 s. This short time results from a flow-based analysis that reduced the traffic to DNS traffic only where the packets are aggregated every 5 s.

Furthermore, the results for the mixed dataset show that the PART classifier extracted rule model has a 99.95% accuracy rate and a 3.84% false-positive rate, which outperformed the JRip classifier. Moreover, the proposed hybrid model achieved even better detection accuracy at 99.96% with only a 1.6% FP rate, which surpassed the other extracted models. In contrast, the F1 score and precision were the same in value.

As for the NIMS-based dataset results, the PART-extracted model also outperformed the JRip-extracted model’s accuracy rate. Similarly, the proposed hybrid model has a 99.97% accuracy rate and a 5% FP rate, which is better than PART and JRip extracted models.

The FP rate for the NIMS-based dataset was higher compared to the result of the mixed dataset. As mentioned in the previous section, the NIMS-based dataset contains fewer records of normal traffic instances, leading to a biased detection rule. Consequently, the result shows a higher FP rate than the mixed dataset, which contains a higher number of normal traffic instances. Hence, having a higher percentage of normal instances in a training dataset is imperative for machine learning classifier training to develop more accurate extracted detection rules with a low FP rate.

Furthermore, the high detection accuracy rate is due to the evaluation of the detection model using a 10-fold cross-validation testing method where the testing data is the same as in trained data. The detection accuracy rate could be reduced if the detection model evaluated using a real-world or supplied dataset. In addition, the data pre-processing, which is the first stage of the proposed approach, has contributed to the enhancing of the detection accuracy

Since high accuracy and low FP rates are essential for botnet detection, the evaluation results for both datasets guarantee the suitability of the proposed hybrid rule model to detect DNS-based botnet with the best accuracy and FP rate of the mixed dataset.

### Result comparison

Haddadi et al. (2014) proposed an approach for botnet detection and tested its performance against NIMS dataset (Haddadi et al., 2014). Later research conducted by the same researchers (Haddadi et al., 2014) used two methods during the pre-processing stage: (1) without using hypertext transfer protocol (HTTP) filters; and (2) using HTTP filters. The first method yielded an 87.5% botnet detection accuracy, while the second method obtained 91.5% accuracy. However, since our proposed approach was not using HTTP filters, we only compared our results with the first test case (Haddadi et al., 2014). Table 11 shows the comparison results.

**Table 11:** Comparison of proposed approach with Haddadi el al. (2014).

Dataset	Proposed approach		Haddadi et al. (2014)	
	Accuracy	FP Rate	Accuracy	FP Rate
NIMS	99.97%	**5%**	87.5%	13.25%
MIXED	99.96%	1.6%	–	–

Like our methodology, Deepbot (Shi & Sun, 2020) also used a hybrid model. It utilised RNN and LSTM algorithms to extract hybrid models for botnet traffic classification. However, despite extracting only 11 DNS features compared to 35 network traffic features by Shi & Sun (2020), our study obtained a better result (99.96% *vs.* 99.36%) with a higher F1 score of 99.97% vs. 98.4%. Table 12 shows the comparison results.

**Table 12:** Comparison of proposed approach with deepbot (Shi & Sun, 2020).

Proposed approach		Deepbot (Shi & Sun, 2020)	
Accuracy	F1	Accuracy	F1
99.96%	99.97%	99.36%	98.4%

The proposed new enriched DNS features computed with the aid of information theory contributed to a higher accuracy rate. However, as discussed earlier, the low number of normal instances led to an FP rate of 5% for the NIMS dataset. Thus, to reduce the FP rate, the study used a mixed dataset that comprised a higher percentage of normal instances and successfully achieved a lower FP rate (1.6%).

## Conclusion

Nowadays, botnets are more diverse, resilient, widespread, and utilised in many cyber attacks. Therefore, there is a pressing need for a better botnet detection method. This study presents a hybrid rule-based approach for detecting DNS-based botnet. New features are proposed and used to form new rules. A total of 32 rules extracted using PART and JRip machine learning algorithms are used to detect DNS-based botnets in the datasets. The performance of the proposed approach was evaluated using two benchmark datasets (NIMS and CTU13). The experimental results show that the detection accuracy of the proposed approach achieved 99.97% and 99.96% for NIMS and mixed datasets, respectively. Meanwhile, the FP rates are 5% and 1.6% for NIMS and mixed datasets, respectively. The comparison results show that our proposed approach outperformed other existing approaches.

Finally, this research opens avenues for future research in the following aspects: (i) adapting the proposed rules to detect blockchain-based DNS botnets, (ii) hybridising the resulted rules with other approaches, such as the signature-based approach, could improve DNS-based botnet detection accuracy further, (iii) investigating and study the impact of encrypted DNS traffic, such as DoH (DNS-over-HTTPS) and DoT (DNS-over-TLS), on the proposed DNS-based botnet detection approach, and (iv) scaling behaviour analysis to better understand the applicability of the proposed approach in the real world.

## Supplemental Information

10.7717/peerj-cs.640/supp-1 Supplemental Information 1 The Output of JRip Classifier.

10.7717/peerj-cs.640/supp-2 Supplemental Information 2 The Output of the PART Classifier.

10.7717/peerj-cs.640/supp-3 Supplemental Information 3 The Hybrid Detection Ruleset.

10.7717/peerj-cs.640/supp-4 Supplemental Information 4 Dataset and python code.

## Additional Information and Declarations

### Data Availability

The following information was supplied regarding data availability: Python script and the dataset used after cleaning are available in the Supplemental Files.
