# Supplementary material for: Hybrid rule-based botnet detection approach using machine learning for analysing DNS traffic
Source: PeerJ Comput Sci. 2021 Aug 13;7:e640. doi: 10.7717/peerj-cs.640 (PMC8372004; doi:10.7717/peerj-cs.640)
Supplement: Supplemental Information 1 [file peerj-cs-07-640-s001.docx]

**Appendix A1. The output of JRip Classifier**

To build the final hybrid detection model and as illustrated in Fig. 14. The model ruleset is inducted using the two training datasets described in Section 4 as JRip input. The classifier entity implemented using the Weka machine learning tool. Therefore, there are two ruleset mode outputs.

1. **The mixed dataset output of the JRip classifier is:**

| === Run information === | | |
| --- | --- | --- |
| Scheme | : | weka.classifiers.rules.JRip -F 3 -N 2.0 -O 2 -S 1 |
| Relation | : | mix 100-weka.filters.unsupervised.attribute.Remove-R1 |
| Instances | : | 45202 |
| Attributes | : | 11 |
|  | : | NO_Distinct_Destination |
|  | : | Arg_TTL |
|  | : | NO_Distinct_TTL |
|  | : | NO_Distinct_Packet |
|  | : | No_suc_resp |
|  | : | No_error_resp |
|  | : | Avg_domain_ent |
|  | : | Ratio_suc_resp |
|  | : | rand_query |
|  | : | number_record_type |
|  | : | Class |
| Test mode | : | 10-fold cross-validation |

**=== Classifier model (full training set) ===**

**JRip rules:**

================

| (No_error_resp <= 1) and (NO_Distinct_TTL >= 2) and (No_suc_resp <= 7) and (Avg_domain_ent <= 3.555269) => class=normal (347.0/0.0) |
| --- |
| (No_error_resp <= 3) and (NO_Distinct_TTL >= 4) => class=normal (166.0/2.0) |
| (No_error_resp <= 7) and (Arg_TTL >= 54) and (Ratio_suc_resp >= 0.875) and (Arg_TTL <= 1308.111111) and (NO_Distinct_TTL >= 2) and (number_record_type >= 2) and (Ratio_suc_resp <= 1.142857) => class=normal (30.0/0.0) |
| (No_error_resp <= 1) and (number_record_type >= 2) and (No_suc_resp <= 2) and (Arg_TTL >= 1) => class=normal (32.0/1.0) |
| (No_error_resp <= 5) and (number_record_type >= 2) and (No_suc_resp <= 1) => class=normal (9.0/1.0) |
| (No_error_resp <= 3) and (Arg_TTL >= 116) and (Avg_domain_ent <= 3.103632) and (Arg_TTL <= 900) => class=normal (8.0/0.0) |
| (No_error_resp <= 3) and (NO_Distinct_TTL >= 2) and (Arg_TTL <= 4571.916667) and (NO_Distinct_Packet >= 3) and (rand_query >= 1.521928) => class=normal (7.0/2.0) |
| (No_error_resp <= 1) and (Ratio_suc_resp <= 0.5) and (NO_Distinct_Destination <= 1) and (NO_Distinct_Destination >= 1) => class=normal (23.0/9.0) |
| (No_error_resp <= 7) and (Arg_TTL >= 299) and (No_suc_resp <= 2) and (Ratio_suc_resp >= 1) => class=normal (4.0/0.0) |
| => class=attack (44576.0/14.0) |
|  |
| Number of Rules : 10 |
|  |
|  |
| Time taken to build model: 5.23 seconds |

======================

**2. The NIMS dataset output of the JRip classifier is:**

| === Run information === | | |
| --- | --- | --- |
| Scheme | : | weka.classifiers.rules.JRip -F 3 -N 2.0 -O 2 -S 1 |
| Relation | : | nims 100 |
| Instances | : | 44677 |
| Attributes | : | 12 |
|  |  | Source_IP |
|  |  | NO_Distinct_Destination |
|  |  | Arg_TTL |
|  |  | NO_Distinct_TTL |
|  |  | NO_Distinct_Packet |
|  |  | No_suc_resp |
|  |  | No_error_resp |
|  |  | Avg_domain_ent |
|  |  | Ratio_suc_resp |
|  |  | rand_query |
|  |  | number_record_type |
|  |  | Class |
| Test mode | : | 10-fold cross-validation |

=== Classifier model (full training set) ===

JRip rules:

=======================

| (Ratio_suc_resp >= 0.875) and (Avg_domain_ent <= 3.190809) and (rand_query >= 1.521928) and (No_error_resp <= 6) => class=normal (64.0/0.0) | | |
| --- | --- | --- |
| (No_error_resp <= 0) and (number_record_type >= 2) and (NO_Distinct_Packet >= 4) and (Avg_domain_ent <= 3.282302) => class=normal (17.0/0.0) | | |
| (No_error_resp <= 0) and (number_record_type >= 2) and (Avg_domain_ent <= 3.359419) and (Arg_TTL <= 900) => class=normal (25.0/10.0) | | |
| (Ratio_suc_resp >= 0.75) and (Avg_domain_ent <= 2.788763) => class=normal (3.0/1.0) | | |
| => class=attack (44568.0/2.0) | | |
|  | | |
| Number of Rules | : | 5 |
|  | | |
|  | | |
| Time taken to build model | : | 1.34 seconds |
