# Supplementary material for: Hybrid rule-based botnet detection approach using machine learning for analysing DNS traffic
Source: PeerJ Comput Sci. 2021 Aug 13;7:e640. doi: 10.7717/peerj-cs.640 (PMC8372004; doi:10.7717/peerj-cs.640)
Supplement: Supplemental Information 2 [file peerj-cs-07-640-s002.docx]

**Appendix A2. The Output of the PART Classifier**

Before selecting the final detection model, the PART classifier is used to extract the second detection ruleset using the two training datasets described in Section 4 as classifier input, as illustrated in Fig. 14. Thus, there are two outputs.

1. **The mixed dataset output for the PART classifier is:**

| === Run information === | | |
| --- | --- | --- |
| Scheme | : | weka.classifiers.rules.PART -M 2 -C 0.25 -Q 1 |
| Relation | : | mix 100-weka.filters.unsupervised.attribute.Remove-R1 |
| Instances | : | 45202 |
| Attributes | : | 11 |
|  |  | NO_Distinct_Destination |
|  |  | Arg_TTL |
|  |  | NO_Distinct_TTL |
|  |  | NO_Distinct_Packet |
|  |  | No_suc_resp |
|  |  | No_error_resp |
|  |  | Avg_domain_ent |
|  |  | Ratio_suc_resp |
|  |  | rand_query |
|  |  | number_record_type |
|  |  | Class |
| Test mode | : | 10-fold cross-validation |

=== Classifier model (full training set) ===

PART decision list

------------------

| NO_Distinct_TTL <= 1 AND | | |
| --- | --- | --- |
| NO_Distinct_Packet > 1 AND | | |
| Ratio_suc_resp <= 0.857143 AND | | |
| No_error_resp > 5: attack (43282.0) | | |
| NO_Distinct_TTL > 2 AND | | |
| No_error_resp <= 1 AND | | |
| NO_Distinct_TTL > 3: normal (279.0/2.0) | | |
| No_error_resp > 0 AND | | |
| NO_Distinct_TTL <= 2 AND | | |
| No_suc_resp > 2: attack (397.0) | | |
| NO_Distinct_TTL <= 1 AND | | |
| number_record_type <= 1 AND | | |
| Arg_TTL <= 294.716667 AND | | |
| Ratio_suc_resp <= 0.75 AND | | |
| No_error_resp > 0 AND | | |
| rand_query <= 1.247556: attack (290.0) | | |
| NO_Distinct_TTL <= 1 AND | | |
| number_record_type <= 1 AND | | |
| Arg_TTL <= 294.716667 AND | | |
| No_suc_resp > 0: attack (283.0/1.0) | | |
| NO_Distinct_TTL > 1 AND | | |
| No_suc_resp <= 4 AND | | |
| Ratio_suc_resp > 0.533333: normal (239.0) | | |
| NO_Distinct_Destination > 2 AND | | |
| Ratio_suc_resp <= 0.487179: attack (104.0) | | |
| number_record_type <= 2 AND | | |
| No_error_resp <= 7 AND | | |
| Avg_domain_ent > 3.324112 AND | | |
| number_record_type > 0 AND | | |
| No_suc_resp > 3 AND | | |
| NO_Distinct_TTL <= 3 AND | | |
| No_error_resp <= 1 AND | | |
| Avg_domain_ent > 3.440818: attack (42.0) | | |
| number_record_type > 2: attack (42.0) | | |
| No_error_resp <= 7 AND | | |
| number_record_type <= 0 AND | | |
| Avg_domain_ent > 3.441374: attack (30.0/1.0) | | |
| No_error_resp > 7: attack (28.0/1.0) | | |
| Arg_TTL <= 21600 AND | | |
| NO_Distinct_Packet > 3 AND | | |
| Arg_TTL <= 7147: normal (27.0/1.0) | | |
| No_suc_resp <= 1 AND | | |
| NO_Distinct_TTL > 0: normal (22.0) | | |
| Arg_TTL > 449.875 AND | | |
| number_record_type > 1 AND | | |
| Avg_domain_ent > 2.805876 AND | | |
| NO_Distinct_TTL <= 1: attack (24.0) | | |
| NO_Distinct_Packet <= 3 AND | | |
| No_suc_resp > 7 AND | | |
| Avg_domain_ent > 2.831039 AND | | |
| NO_Distinct_Destination > 1: attack (8.0) | | |
| NO_Distinct_Packet <= 3 AND | | |
| No_error_resp > 0 AND | | |
| NO_Distinct_Destination <= 1: attack (12.0) | | |
| NO_Distinct_Packet <= 3 AND | | |
| No_error_resp <= 1 AND | | |
| NO_Distinct_TTL > 0 AND | | |
| No_suc_resp <= 3: normal (22.0) | | |
| NO_Distinct_Packet <= 3 AND | | |
| No_error_resp <= 1 AND | | |
| rand_query <= 1.666056 AND | | |
| NO_Distinct_Destination <= 1 AND | | |
| Ratio_suc_resp <= 0.125: normal (21.0/6.0) | | |
| Avg_domain_ent > 2.893186 AND | | |
| No_error_resp <= 2 AND | | |
| No_error_resp <= 1 AND | | |
| NO_Distinct_Destination <= 1 AND | | |
| NO_Distinct_TTL <= 2 AND | | |
| Ratio_suc_resp <= 1.054054 AND | | |
| NO_Distinct_Packet <= 2: attack (16.0/2.0) | | |
| NO_Distinct_Packet <= 3 AND | | |
| NO_Distinct_Packet > 1 AND | | |
| Avg_domain_ent <= 3.361146 AND | | |
| Arg_TTL <= 1969.230769: normal (17.0) | | |
| Ratio_suc_resp <= 1.1 | : | attack (14.0) |
|  | : | normal (3.0/1.0) |
|  | | |
| Number of Rules | : | 22 |
|  | | |
|  | | |
| Time taken to build model |  | 0.8 seconds |

**2. The NIMS dataset output for the PART classifier is:**

| === Run information === | | |
| --- | --- | --- |
| Scheme | : | weka.classifiers.rules.PART -M 2 -C 0.25 -Q 1 |
| Relation | : | nims 100 |
| Instances | : | 44677 |
| Attributes | : | 12 |
|  |  | Source_IP |
|  |  | NO_Distinct_Destination |
|  |  | Arg_TTL |
|  |  | NO_Distinct_TTL |
|  |  | NO_Distinct_Packet |
|  |  | No_suc_resp |
|  |  | No_error_resp |
|  |  | Avg_domain_ent |
|  |  | Ratio_suc_resp |
|  |  | rand_query |
|  |  | number_record_type |
|  |  | Class |
| Test mode | : | 10-fold cross-validation |

=== Classifier model (full training set) ===

PART decision list

------------------

| NO_Distinct_TTL <= 2 AND | | |
| --- | --- | --- |
| Ratio_suc_resp <= 0.727273 AND | | |
| No_error_resp > 0: attack (44003.0) | | |
| NO_Distinct_TTL <= 3 AND | | |
| number_record_type <= 1: attack (405.0) | | |
| NO_Distinct_TTL > 3 AND | | |
| No_error_resp <= 10 AND | | |
| NO_Distinct_Packet > 4: normal (52.0) | | |
| No_error_resp > 1: attack (69.0) | | |
| Avg_domain_ent > 3.307406 AND | | |
| NO_Distinct_Destination <= 1 AND | | |
| NO_Distinct_Packet <= 2: attack (38.0/1.0) | | |
| Avg_domain_ent <= 3.440818 AND | | |
| NO_Distinct_Packet > 3 AND | | |
| Avg_domain_ent <= 3.305419: normal (24.0) | | |
| Avg_domain_ent > 2.828196 AND | | |
| No_suc_resp > 3 AND | | |
| Arg_TTL > 576: attack (47.0/1.0) | | |
| NO_Distinct_TTL > 0 AND | | |
| Avg_domain_ent <= 3.451193 AND | | |
| NO_Distinct_Destination <= 2 AND | | |
| rand_query <= 1.501614: normal (16.0) | | |
| NO_Distinct_TTL <= 1: attack (17.0/1.0) | | |
|  | : | normal (6.0/1.0) |
|  | | |
| Number of Rules | : | 10 |
|  | | |
|  | | |
| Time taken to build model | : | - 1. seconds |
