# Supplementary material for: Hybrid rule-based botnet detection approach using machine learning for analysing DNS traffic
Source: PeerJ Comput Sci. 2021 Aug 13;7:e640. doi: 10.7717/peerj-cs.640 (PMC8372004; doi:10.7717/peerj-cs.640)
Supplement: Supplemental Information 3 [file peerj-cs-07-640-s003.docx]

**Appendix A3. The hybrid Detection Ruleset**

The Hybrid detection ruleset is the union of the output from both JRip (Appendix A1) and PART classifier (Appendix A2). Moreover, the mixed dataset contains more normal instances, which leads to more accurate extracted rules. Thus, the final rule is a set of 32 hybrid rules built using a mixed dataset.
